# Conserved and lineage-specific mechanisms drive chromatophore differentiation in reptiles

**DOI:** 10.1038/s41467-026-75281-5

**Published:** 2026-07-06

**Authors:** Pierre-Yves Helleboid, Athanasia C. Tzika

**Affiliations:** Laboratory of Artificial & Natural Evolution (LANE), Department of Genetics & Evolution, University of Geneva, Geneva, Switzerland

**Keywords:** Differentiation, Non-model organisms, Ectoderm, Evolutionary developmental biology

## Abstract

The mechanisms by which novel differentiation pathways evolve to produce new cell types are still not fully understood. Chromatophores, the pigmented cells in the skin, offer an ideal paradigm because each type independently develops from neural crest cells to produce a distinct colour using well-characterised biosynthetic pathways. Here we show, using single-cell gene expression analyses, that canonical chromatophores develop in the embryonic skin of corn snakes and bearded dragon lizards. Yet, we identify previously undescribed chromatophore subtypes in the bearded dragon. These populations co-express progenitor and mature markers and possibly contribute to embryonic skin patterning, as revealed by whole-mount in situ hybridisation. Comparative analyses uncover that while mature chromatophores show cross-species similarity reflecting shared pigmentary function, progenitor states differ in transcription factor usage, including species-specific deployment of MITF, PAX7, and TFEC. Integration with teleost and amphibian datasets confirms that diversification of pigmentation arises through distinct progenitor trajectories converging on similar mature states.

## Abstract (web summary)

Gene expression analysis of pigment cells in snake and lizard embryos and comparison with fish and frog shows that progenitors follow different developmental paths in each species yet mature cells preserve core functions. Previously unknown cell types are also uncovered.

## Introduction

During development, a large set of diverse cell types differentiates from multipotent progenitors. Cell fate is progressively defined by the activation of alternative transcriptional cascades, and upon maturation, cells express type-specific genes appropriate for their function. Recent advances in single-cell transcriptomics enable dissection of these processes with remarkable resolution and elucidate the mechanisms that govern the transcriptional wiring of the differentiating cells during embryogenesis^1^.

Chromatophores are neural crest-derived cells that produce pigments. During development, their progenitors differentiate while migrating and interacting with each other and their environment. Their state and spatial organisation generate a vast array of coloration patterns in the vertebrate skin, which play a critical role in the individual’s survival, including mate choice, predator avoidance, and signalling toxicity^2^. The three main types of chromatophores found in fish, amphibians, and non-avian reptiles are melanophores, xanthophores, and iridophores, which generate black/brown, yellow/red, and structural coloration, respectively^3^.

Reptilian skin coloration is strikingly diverse, ranging from cryptic to conspicuously bright and can even change over short and long-time scales^4,5^. Yet the developmental mechanisms responsible for these traits remain largely unexplored. Reptiles are separated from ray-finned fishes and amphibians by more than 400 and 350 million years of evolution, respectively, and unlike many teleosts and amphibians, they lack post-embryonic metamorphosis^6^. Consequently, reptilian chromatophores differentiate during embryogenesis and directly establish the skin pattern of the hatchlings. Indeed, in most colour-changing species, the same cell types persist and only their state changes.

**Fig. 1:**
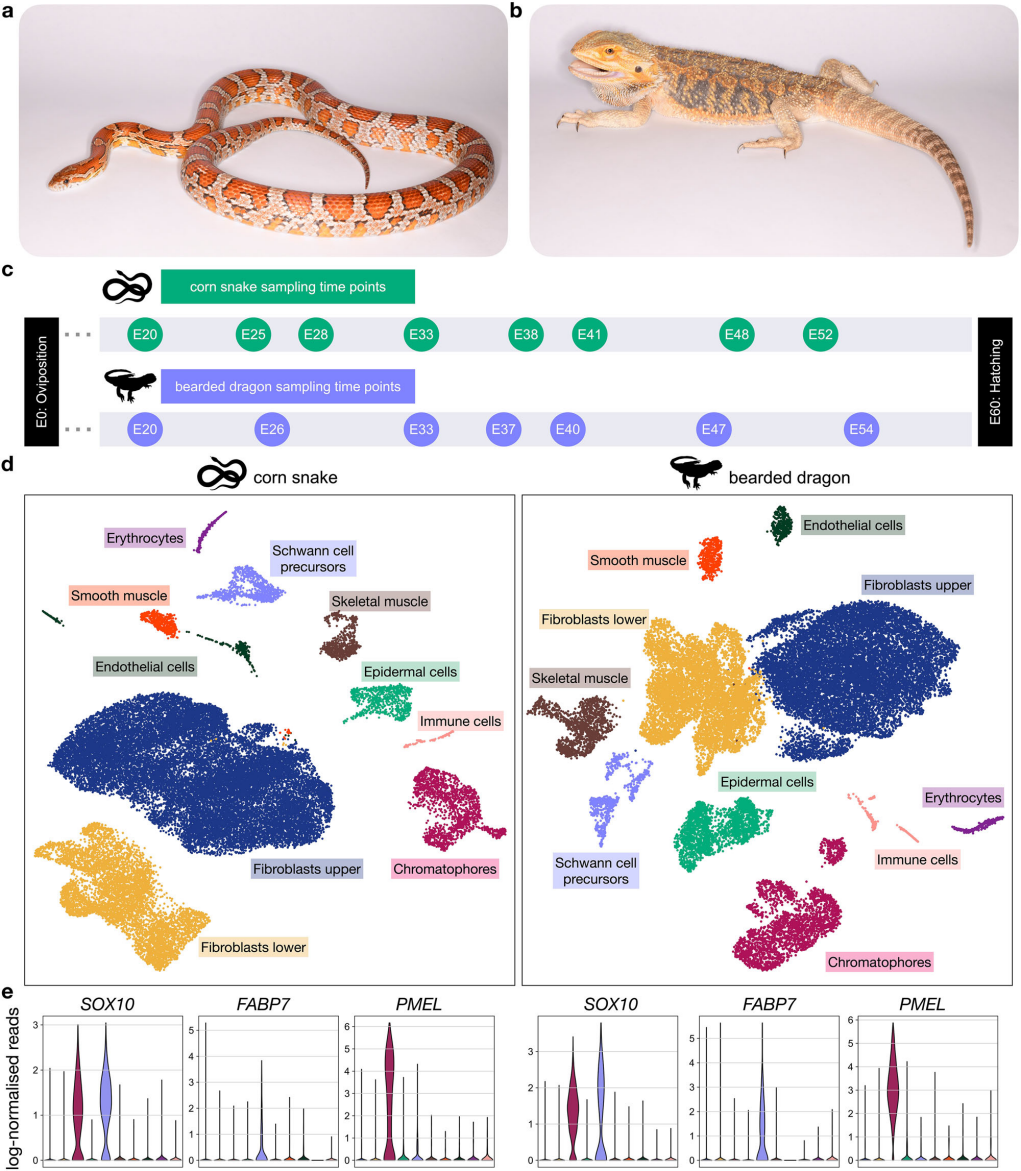
Cell types in the embryonic skin of corn snakes and bearded dragons. Photograph of an adult corn snake (**a**) and an adult bearded dragon (**b**). **c** Sampling timepoints in embryonic days (E) for the two species. **d** UMAP plots with cell populations coloured by cell type. **e** Violin plots of *SOX10*, *FABP7* and *PMEL* expression colour-coded as in panel d. *FABP7* encodes the Fatty Acid-Binding Protein 7, enriched in Schwann cell precursors. Pictograms were downloaded from PhyloPic and are freely available under the CC0 1.0 Universal license.

Here, we present single-cell transcriptomic analyses of chromatophore development in two squamate species, the corn snake (*Pantherophis guttatus*) and the bearded dragon (*Pogona vitticeps*), which diverged 161 MYA^7^ (Fig. 1a, b). Both species are amenable to developmental studies in a laboratory setting^8^ and established models for investigating skin coloration and skin appendages development in snakes and lizards^9–16^. They display elaborate skin coloration patterns, yet little is known about the dynamic processes that generate them. We identify canonical pigment cell types in both, as well as previously undescribed chromatophore types in the bearded dragon and proceed to reconstruct their differentiation trajectories. We further investigate the expression profile of key transcription factors and their involvement in cell type specification. Indeed, key transcription factors known to regulate chromatophore development in teleosts, such as MITF, PAX7, and TFEC, remain poorly characterized in reptiles^17–19^. Integration with chromatophore datasets from zebrafish and frog, two metamorphic species, reveals both conserved and novel transcriptional programs. These findings elucidate how lineage-specific innovations emerge and illuminate both the universality and the evolutionary flexibility of differentiation mechanisms. Furthermore, our results show that evolutionary changes in early differentiation can create diverse cell types that converge on similar mature functions.

## Results

### Cell types in the embryonic skin of corn snakes and bearded dragons

Following oviposition, corn snake and bearded dragon eggs are incubated for 60 days before hatchlings emerge with a fully-formed skin coloration pattern. The dorsal skin of corn snakes is characterised by red blotches on an orange background (Fig. 1a). Bearded dragons display two light-coloured lines at each side of the dorsal midline and alternating dark brown and light-coloured bands at their flanks. The intensity of the light bands is, in general, greater in hatchlings, and their hue may vary among individuals from light brown to yellow or red (Fig. 1b).

We performed single-cell RNA-sequencing (scRNA-seq) on corn snake and bearded dragon embryonic dorsal skin collected at developmental time points spanning the differentiation and maturation of chromatophores (Fig. 1c, Supplementary Figs. 1, 2, Supplementary Data 1). Cluster assignment, presented on Uniform Manifold Approximation and Projection (UMAP) plots, was based on cell type-specific markers (Fig. 1d, Supplementary Fig. 3). Chromatophores expressed the neural crest cell marker *SOX10*; they could be distinguished from Schwann cell precursors^20^, expressing *FABP7*, by the specific expression of *PMEL* (Fig. 1e).

### Corn snake chromatophores express established lineage markers

**Fig. 2:**
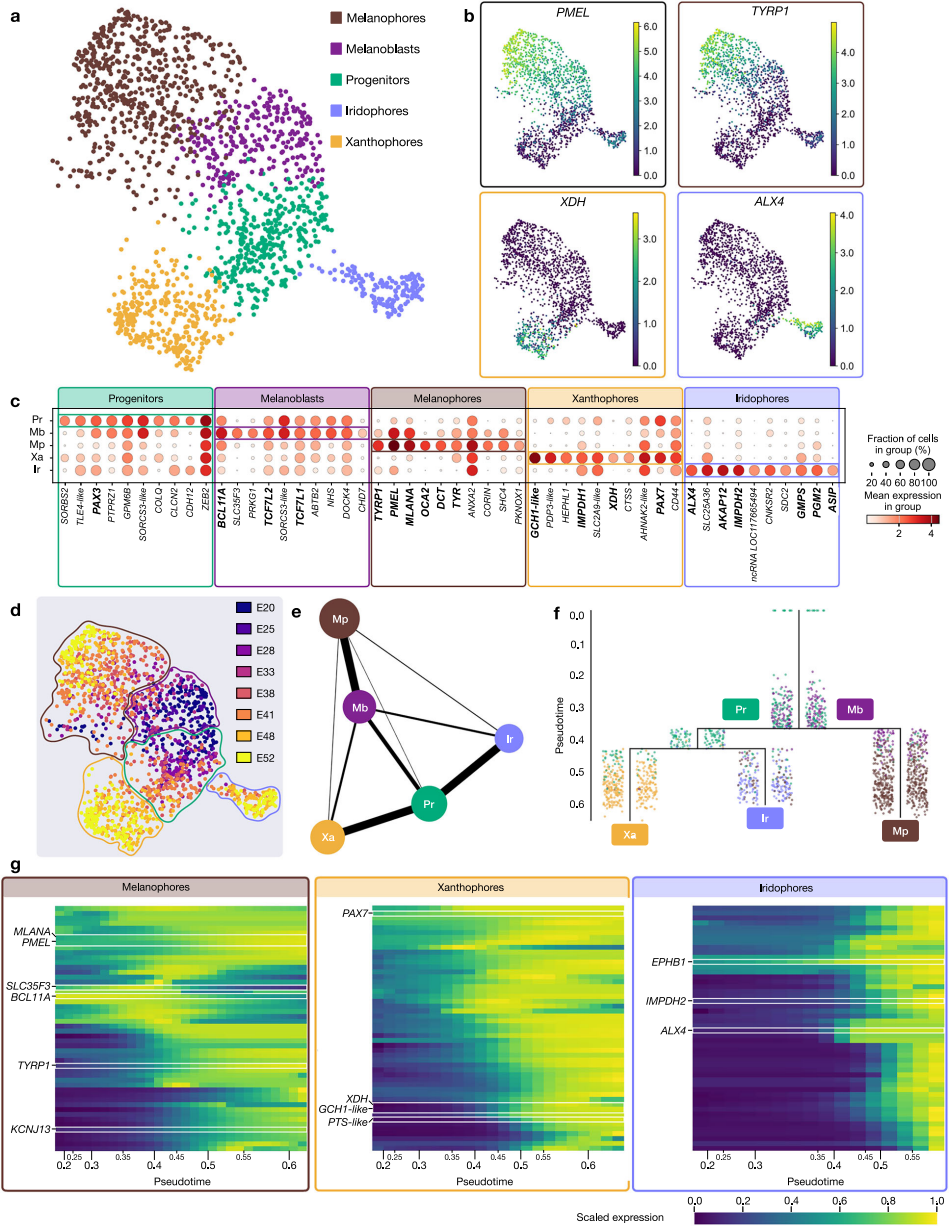
Corn snake chromatophores express established lineage markers. **a** UMAP of chromatophores colour-coded by lineage identity. **b** UMAPs showing the expression (log-normalised reads) of key chromatophore lineage markers. **c** Dot plot representing the average expression and percentage of expressing cells of the ten most differentially expressed markers of each cluster. Differential expression was computed using a two-sided Wilcoxon rank-sum test. (Pr: Progenitors; Mb: Melanoblasts; Mp: Melanophores; Xa: Xanthophores; Ir: Iridophores). *PDP3-like* corresponds to *Putative Defence Protein 3-like*. *LOC117665494* is a non-coding gene. **d** UMAP of chromatophores colour-coded according to the embryonic day sampled. The colour-coded outlines encompass the chromatophore cell types as defined in (**a**). **e** PAGA correlation graph of the corn snake chromatophore clusters. Edge weights represent the statistical measure of inter-cluster connectivity computed by PAGA. **f** Trajectory tree inferred by URD, tracing the progression from the root population to terminally differentiated melanophores, iridophores, and xanthophores. Cells are coloured by lineage and positioned along the y-axis according to their assigned pseudotime. Branch points were determined using the preference divergence method with a significance threshold of *p* < 0.001. **g** Gene expression cascades of the top 50 differentially expressed genes, ranked by their URD-derived AUCPR.all values, along the melanophore, xanthophore, and iridophore differentiation trajectories. Expression is shown as a moving-window average in pseudotime (x-axis) scaled to the maximum observed expression. Note that the pseudotime axis is unevenly spaced: bins contain a fixed number of cells, so regions with more cells have more bins. Selected marker genes are indicated on the y-axis and their dynamic expression is highlighted with white rectangles.

In the corn snake, we identified three cell clusters corresponding to mature chromatophores, namely melanophores, xanthophores and iridophores (Fig. 2a). These expressed established markers, such as *TYRP1*, encoding an enzyme essential for melanin synthesis in melanophores, *XDH*, encoding a pteridine synthesis enzyme in xanthophores, and the expression of the transcription factor *ALX4* in iridophores, respectively (Fig. 2b, c)^21–23^. We also found distinct clusters of melanoblasts and chromatophore progenitors. Note that the assignment to the latter cluster was based on the shared transcriptional features with pigment cells and the expression of progenitor-associated marker genes; we cannot exclude that these cells have already engaged in differentiation, rather than being fully competent progenitors.

Based on differential gene expression analyses (Fig. 2c, Supplementary Data 2), melanophores were characterised by the expression of genes associated with melanogenesis, such as *MLANA*, *OCA2*, *DCT*, and *TYR*^23^. We have previously shown that *PMEL*, coding for the amyloid fibrils structuring melanosomes, is widely expressed by early-stage chromatophores^13^. Here, we confirm high expression levels of *PMEL* in both melanophores and melanoblasts, yet the expression of terminal differentiation markers, such as *TYR* and *TYRP1*, is restricted to melanophores. Attesting to the precursor status of melanoblasts is the fact that they share with the progenitors cluster the expression of transcription factors known to be involved in neural progenitor biology, such as *BCL11A*^24^, *TCF7L2*^25^, and *TCF7L1*^26^, consistent with their neural crest origin.

Along with *XDH*, xanthophores differentially expressed *GCH1*, another enzyme involved in the pteridine biosynthesis pathway^27^. The transcription factor *PAX7* is essential for the maturation of xanthophores in the zebrafish^21^ and the differentiation of xanthophores and iridophores in the leopard gecko^28^. In the corn snake, it is expressed by the progenitor cells and at lower levels by the melanoblasts, but its strong expression persists in the xanthophore lineage until maturation. Progenitor cells also express *PAX3*, another transcription factor that regulates the differentiation of chromatophore progenitor cells in teleost fish^29^. *IMPDH1* encodes a rate-limiting enzyme involved in the production of guanine nucleotides, which are necessary for the biosynthesis of pteridine and the formation of iridophore reflective guanine crystals^11,30^. Accordingly, we find it expressed in both xanthophores and iridophores, but the expression of its paralog, *IMPDH2*, is restricted to iridophores. The latter cells also express *GMPS*, an enzyme involved in guanine metabolism^31^, and *PGM2* implicated in purine degradation^32^.

### Differentiation dynamics of the corn snake chromatophores

At early developmental stages, we primarily find progenitors and melanoblasts, while differentiated and mature chromatophores appear later (Fig. 2d, Supplementary Fig. 4a) as expected by the macroscopic observation of the embryos and the corresponding cell suspensions (Supplementary Fig. 1). We present a PAGA (Partition-Based Graph Abstraction) graph, where cell clusters are depicted as nodes interconnected by edges weighted based on the statistical confidence in inter-cluster connectivity (Fig. 2e)^33^. The progenitors’ cluster is strongly connected with xanthophores, iridophores and melanoblasts, the later also being connected with melanophores. This connectivity is corroborated by the trajectory analysis performed with URD, a computational tool that reconstructs developmental trajectories as a branching tree^34^. Progenitor cells and melanoblasts co-exist early in pseudotime, with melanophores branching out first, followed by xanthophores and iridophores (Fig. 2f, Supplementary Movie 1). Indeed, this trajectory roughly matches with the developmental stages at which each cell type was sampled (Supplementary Figs. 1, 4a, b).

The expression dynamics in melanophores follows three distinct trends through pseudotime (Fig. 2g): (i) persistent expression throughout differentiation and maturation as seen for *PMEL* and *MLANA* highlighting their diverse functions during development, (ii) high expression levels at the onset of differentiation that gradually reduce as for the transcription factor *BCL11A* and the melanoblast-specific marker *SLC35F3*, and (iii) progressively increasing levels during maturation as observed for *TYRP1* and *KCNJ13*, with the latter known to regulate chromatophore interactions in zebrafish^35^. In xanthophores, we observe only two trends, either persistent expression as seen for *PAX7* or gradually increasing expression as for the pteridine-synthesis enzymes *XDH*, *GCH1* and a *PTS* paralog. No iridophore markers are expressed at early stages, implying that iridophore determination requires activation of a distinct transcriptional program at later developmental stages. The iridophore specifying transcription factor *ALX4*^35^ and *EPHB1*, a tyrosine kinase receptor playing an important role in axon guidance during brain development as well as synapse formation, are among the first markers expressed^36^. The expression of the guanine-synthesis enzyme *IMPDH2* initiates later.

### Secondary populations of melanophores and xanthophores present in the bearded dragon embryonic skin

**Fig. 3:**
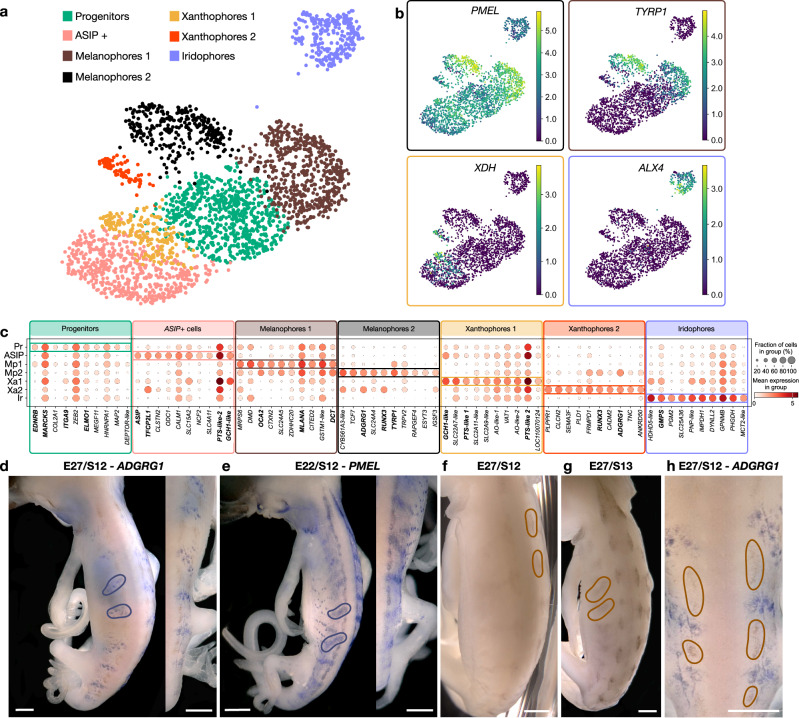
Bearded dragon chromatophore lineage identification. **a** UMAP of chromatophores colour-coded by lineage identity. **b** UMAPs showing the expression (log-normalised reads) of key chromatophore lineage markers. **c** Dot plot representing the average expression and percentage of expressing cells of the 10 most differentially expressed markers of each cluster. Differential expression was computed using a two-sided Wilcoxon rank-sum test. (Pr: Progenitors; As: ASIP+ cells; Mb: melanoblasts; Mp1: melanophores 1; Mp2: melanophores 2; Xa1: xanthophores 1; Xa2: xanthophores 2; Ir: iridophores). **d** Lateral (left) and dorsal (right) view of a bearded dragon embryo stained with a *ADGRG1* probe. **e** Lateral (left) and dorsal (right) view of the bearded dragon embryo stained with a *PMEL* probe. In **d** and **e**, two dorsal stained bands have been highlighted with a blue outline. **f, g** Dorsal view of bearded dragon embryos with visible melanin pigmentation. **h**. Higher magnification of d with the melanin pigmentation highlighted. In **f**, **g**, and **h**, the patches of melanin pigmentation are highlighted with a brown outline. Scale bars: 1 mm.

Similarly to the corn snake, unsupervised clustering of the bearded dragon chromatophores revealed the presence of progenitors, melanophores, xanthophores and iridophores (Fig. 3a,b,c, Supplementary Fig. 4c and Supplementary Data 2). At the earliest developmental stage over 95% of cells are assigned to the progenitors’ cluster (Supplementary Fig. 4c). This is primarily defined by the differential expression of genes associated with the early migratory phase of the chromatophore progenitors from the neural crest, such as *EDNRB*, *MARCKS*, *ITGA9*, and *ELMO1* for cell motility^37–40^. As progenitors share the expression of lineage markers with the other differentiated clusters, such as a *PTS* paralog (*PTS-like-2*) with the xanthophores, *MLANA* and *DCT* with the melanophores, and *GMPS* with the iridophores, they might have actually initiated differentiation, instead of being fully competent progenitors.

Indeed, the primary populations of melanophores, xanthophores and iridophores all express established markers for each lineage (Fig. 3c). Although a unique iridophore population is detected, there are two subpopulations for melanophores and xanthophores. The two melanophore populations (1 and 2) share comparable levels of expression of canonical melanophore markers, including *OCA2*, *DCT*, and *TYRP1*, and at most stages sampled, the number of melanophores 1 is greater than melanophores 2 (Supplementary Fig. 4c). The relative proportion of xanthophores 2 is also smaller than xanthophores 1 at most stages, and we observe lower expression of the pteridine-synthesis enzymes *GCH1* and *PTS* compared to xanthophores 1.

Both melanophores 2 and xanthophores 2—hereafter referred to as secondary chromatophores—are characterised by the upregulation of *RUNX3* (Runt-related transcription factor 3) and *ADGRG1* (Adhesion G Protein-Coupled Receptor G1) (Fig. 3c). *RUNX3* promotes the transdifferentiation of melanophores into melanoleucophores in the zebrafish dorsal and caudal fins by inducing melanin degradation and inhibiting production of new melanin^41^. Although melanoleucophores have not been described in reptiles thus far, in the bearded dragon secondary populations, *RUNX3* might also inhibit pigment production. *ADGRG1* encodes a G protein-coupled receptor found in the nervous system, specifically in neurons, oligodendrocyte progenitor cells and accompanying Schwann cells, as well as on the plasma membrane of muscle cells and Natural Killer cells^42^.

In addition to secondary chromatophores, *RUNX3* is expressed by a great number of fibroblasts of the upper dermis and epidermal cells (Supplementary Fig. 5a). On the other hand, *ADGRG1* is primarily expressed by the secondary chromatophores and only weakly by a subpopulation of lower dermis fibroblasts and epidermal cells (Supplementary Fig. 5b). We thus performed whole-mount in situ hybridisations (WISH) using an *ADGRG1* species-specific probe to localise the secondary chromatophores on the embryonic skin. Stained cells form bands on the dorsal side of the embryo (Fig. 3d). This pattern resembles the *PMEL* staining (Fig. 3e) at the same developmental stage, forming bands that will eventually be melanized. Some embryos start producing melanin earlier than others with brown spots visible at each side of the dorsal midline (Fig. 3f) that later expand to bands (Fig. 3g). We performed WISH on such an embryo and observed that *ADGRG1-*expressing cells are located between these melanised spots and not on them (Fig. 3h). Thus, the *PMEL-* and *ADGRG1*-expressing cells are likely to form alternating bands along the embryo flanks.

ADGRG1 is known to inhibit the migration of developing brain neuronal cells through its interaction with collagen type III^42^. In addition, when located at the surface of oligodendrocyte precursors, it interacts with extracellular transglutaminase 2 (TGM2) to promote oligodendrocyte precursor cells' proliferation^43^. We could identify a subcluster of lower dermis fibroblasts strongly expressing both genes (Supplementary Fig. 5c). Therefore, we hypothesise that the migration of the secondary chromatophores could be inhibited by collagen III and their in-situ proliferation could be promoted by TGM2. However, further evidence is needed to confirm this.

Given the continuous presence of the secondary populations until the late stages of development, we assume that they participate in the final skin coloration pattern; yet their exact role remains to be elucidated. The WISH *ADGRG1* staining suggests that they form bands between the dark bands observed in hatchlings, but we cannot confirm that these are pigment-producing cells. The involvement of secondary chromatophores in the coloration patterning is further supported by their differential expression of *CLCN2* (Fig. 3c, Supplementary Data 2). Indeed, we have previously shown that the disruption or downregulation of *CLCN2* results in altered coloration pattern in two corn snakes morphs^10^. Note that in the corn snake dataset, *RUNX3* is primarily expressed by fibroblasts with only a few chromatophores expressing it, whereas the six *ADGRG1* paralogs have variable expression profiles both by fibroblasts and chromatophores (Supplementary Fig. 5d).

### Agouti signalling promotes the ventralisation of the bearded dragon coloration but not of corn snakes

The Agouti-signalling protein (ASIP) is an antagonist of the melanocortin 1 receptor (MC1R) and regulates melanogenesis. In mammals, *ASIP* modulates the production of eumelanin and pheomelanin^44,45^ and is expressed by dermal papilla cells at the base of the hair follicle^46^. In zebrafish, *asip1* plays a ventralisation role in skin coloration by inhibiting the differentiation of melanophores and xanthophores^47^. It is primarily expressed by *bnc2+* hypodermal cells in the body, although lower levels can be detected in a small proportion of melanophores and xanthophores^48^. *bnc2* encodes a zinc finger protein and promotes melanophore and xanthophore development through the upregulation of *kitlga*, *csf1a* and *csf1b*^49^.

**Fig. 4:**
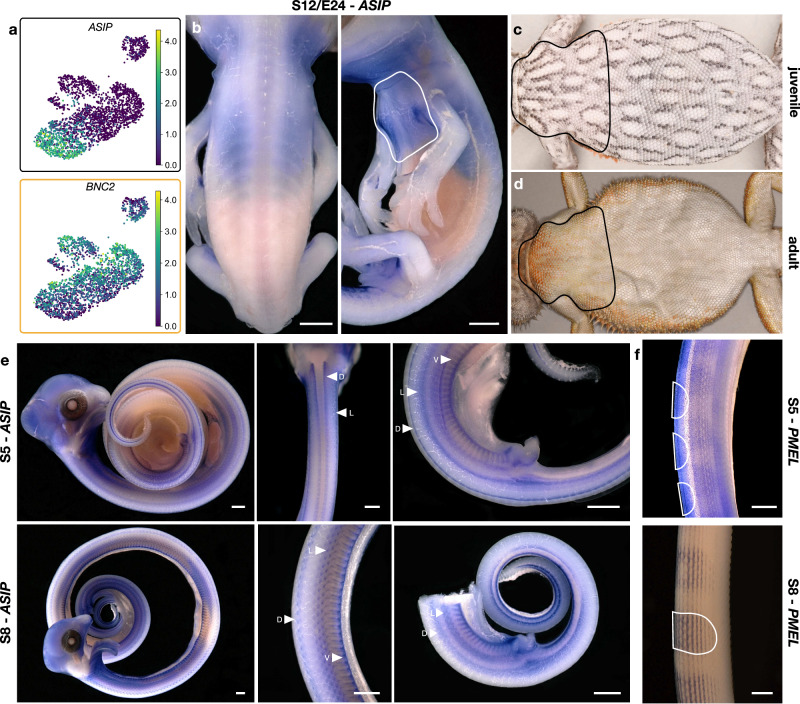
Agouti signalling promotes the ventralisation of the bearded dragon coloration but not of corn snakes. **a** UMAPs showing the expression of *ASIP* and *BNC2* in the bearded dragon dataset. **b** Dorsal (left) and lateral (right) view of bearded dragon embryo stained with a species-specific *ASIP* probe (white outline). Blue staining on the dorsal view corresponds to probe trapping. Ventral view of a juvenile (**c**) and an adult (**d**) bearded dragon individual. The ventral region corresponding to the white outline in (**a**) is outlined in black. **e** WISH with an *ASIP* probe on corn snake embryos. Views of the entire embryo (left), a body part (middle) and the cloaca region (right) are provided. Arrowheads point to the dorsal (D) and lateral (L) lines and the ventral (V) region. **f** Lateral view of corn snake embryos (body part) stained with a *PMEL* probe. Stained aggregates are outlined in white. Scale bars: 1 mm.

In the bearded dragon embryonic skin, a chromatophore *ASIP+* cell population becomes apparent at early stages and persists throughout development albeit in smaller percentages (Supplementary Fig. 4c). *ASIP* is also expressed by a subcluster of lower dermis fibroblasts (Supplementary Fig. 6a). The majority of fibroblasts express *BNC2* (Fig. 4a, Supplementary Fig. 6b,c). Among the differentially expressed genes in the *ASIP+* cluster, the expression of *TFCP2L1*, a transcription factor that maintains the pluripotent state of mouse embryonic stem cells^49^, is shared with the progenitors’ cluster and xanthophores 2, whereas the expression of *GCH1* and *PTS* is shared with xanthophores 1 (Fig. 3c).

We could localise *ASIP*-expressing cells at the ventral side of a bearded dragon embryo (Fig. 4b). Thus, we assume that *ASIP* is expressed ventrally by a cluster of fibroblasts and chromatophore cells, potentially of the xanthophore lineage, contributing to the ventralisation of skin coloration. The *ASIP*+ cells remain undifferentiated, possibly reflecting a stem-like state marked by *TFCP2L1* expression, whereas the iridophores and melanophores fully mature. Indeed, the hatchlings display ventrally a black-and-white pattern composed of iridophores and melanophores (Fig. 4c). As the animal grows melanophores are less visible and some red coloration becomes apparent (Fig. 4d).

In corn snakes, *ASIP* is expressed by iridophores and a great number of upper and lower dermis fibroblasts (Supplementary Fig. 6d). *BNC2* is expressed by most chromatophores, lower dermis fibroblasts and epidermal cells (Supplementary Fig. 6e). WISH with a species-specific *ASIP* probe revealed a complex expression pattern (Fig. 4e). At E16 (S5), we observe a dorsal and a lateral stripe as well as expression in the developing ventral scales. At the same stage, aggregates of *PMEL-*expressing cells start to form (Fig. 4f)^13^. At E22 (S8), the dorsal stripe is maintained, the lateral line is situated at the dorsoventral boundary, and ventrally, the signal is concentrated at the posterior tip of the ventral scales that become asymmetrical. At this stage, the *PMEL* staining corresponds to the dorsal blotches. Therefore, the *ASIP* expression pattern does not correspond to the colouration pattern being established at the same stages. Our findings support a ventralisation role of *ASIP* in the bearded dragon but not the corn snake. Comparative analyses of the dorsal and ventral corn snake skin would be needed to identify the key players that establish such distinct patterns at each side of the animal, and spatial transcriptomics analyses could elucidate the role of *ASIP*.

### Bearded dragon chromatophores differentiate along multiple developmental trajectories

**Fig. 5:**
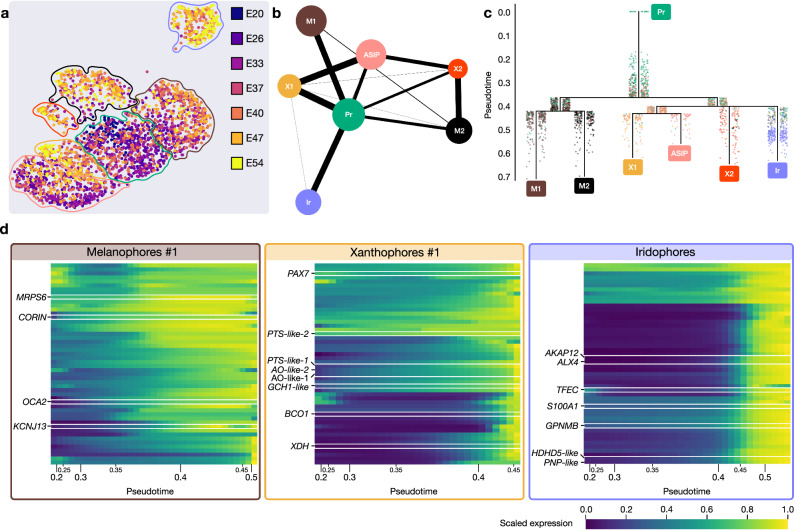
Bearded dragon chromatophore lineages differentiation dynamics. **a** UMAP of chromatophores colour-coded according to the embryonic day sampled. The colour-coded outlines encompass the chromatophore cell types as defined in Fig. 3a. **b** PAGA correlation graph of the bearded dragon chromatophore clusters. Edge weights represent the statistical measure of inter-cluster connectivity computed by PAGA. **c** Trajectory tree inferred by URD tracing the progression from the root population to the mature chromatophores. Cells are coloured by lineage and positioned along the y-axis according to their assigned pseudotime. Branch points were determined using the preference divergence method with a significance threshold of *p* < 0.001. **d** Gene expression cascades of the top 50 differentially expressed genes, ranked by their URD-derived AUCPR.all values, along the melanophore 1, xanthophore 1, and iridophore differentiation trajectories. Expression is shown as a moving-window average in pseudotime (x-axis) scaled to the maximum observed expression. Note that the pseudotime axis is unevenly spaced: bins contain a fixed number of cells, so regions with more cells have more bins. Selected marker genes are indicated on the y-axis and their dynamic expression is highlighted with white rectangles.

In the bearded dragon, the progenitors’ population predominates at the earliest sampled stage, while *ASIP+* cells are represented at later stages at roughly equal proportions as the progenitors. A small number of differentiated melanophores and xanthophores are present already at E20 and their proportion gradually increases, as is the case for iridophores that mature at a later stage (Fig. 5a, Supplementary Fig. 4c). In the PAGA graph (Fig. 5b), the progenitor cells are highly connected with all the other cell types. The *ASIP+* cluster is strongly connected with the two types of xanthophores, suggesting similarities in their transcriptional program. Melanophores 2 and xanthophores 2 have a stronger connection to each other than to their primary counterparts.

The developmental trajectory calculated by URD supports an initial split between the melanophore lineage and that of xanthophores and iridophores (Fig. 5c, Supplementary Movie 2). In the graph, *ASIP+* cells appear as a distinct population closely related to xanthophores 1. The *ASIP* + /xanthophores 1 and 2 closer connection is further supported by the shared *PAX7* expression (Supplementary Fig. 6f, g). For each trajectory, a few early-stage cells are attributed to terminal branches (Supplementary Fig. 4d). These would correspond to a fast-differentiating population of cells that produces pigments early on, as seen in Fig. 3f.

The dynamic expression of melanophore markers through pseudotime differs in bearded dragons and corn snakes (Fig. 5d). In bearded dragons, we observe two categories: (i) genes expressed throughout development, such as *CORIN* known to suppress Agouti signalling in mice^46^ and the less well-studied *MRPS6*^50^, and (ii) genes expressed only at later stages, including *OCA2* and *KCNJ13,* as in the corn snake. Similar trends are observed in xanthophores. For example, *PAX7* and *PTS-like-2* are continuously expressed by this lineage, whereas transcription of the enzymes *GCH1* and *XDH* initiates later. *BCO1*, encoding beta-carotene oxygenase 1, is also expressed by mature xanthophores, suggesting the presence of carotenoids, in addition to pteridines in bearded dragon xanthophores, but not in corn snakes (Supplementary Fig. 7). As in the corn snake, most iridophore markers start to be expressed at the final stages of differentiation.

### Transcription factors involved in chromatophore differentiation

**Fig. 6:**
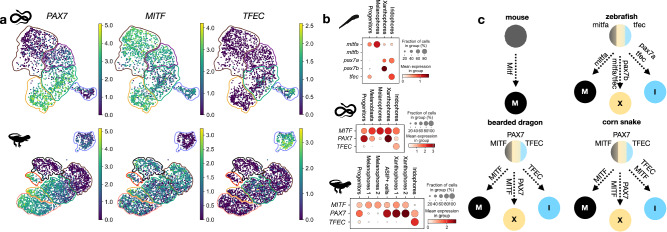
Transcription factors involved in chromatophore differentiation. **a** UMAP plots of *PAX7, MITF* and *TFEC* expression in the corn snake and the bearded dragon embryonic skin. **b** Dot plot summarising the average expression levels and percentage of cells expressing the *PAX7, MITF* and *TFEC* in zebrafish, corn snake and bearded dragon. **c** Schematic illustration of chromatophore differentiation pathways in four vertebrates. Key transcription factors associated with specific lineage trajectories are annotated along the corresponding differentiation paths.

As indicated earlier, *PAX7* is a transcription factor playing an important role in the differentiation of the xanthophore lineage in zebrafish and the xanthophores and iridophores in leopard geckos. In the corn snake, *PAX7* is primarily expressed by the progenitors that will give rise to the xanthophore and iridophore lineages, and it persists in mature xanthophores. In the bearded dragon, *PAX7* expression is shared by the progenitors, the *ASIP*+ cells and the two xanthophore populations. Thus, in both species, its expression clearly distinguishes the xanthophore-related lineages from the others (Fig. 6a). In zebrafish metamorphic stages (Saunders et al. dataset^51^ at 5 days post-fertilisation [dpf] - Fig. 6b), expression of *pax7a* characterises differentiated iridophores and xanthophores. In contrast, *pax7b* is expressed more robustly and specifically in the majority of xanthophores.

Recent studies have emphasised the collaborative role of two additional transcription factors of the MiT family, *MITF* and *TFEC*, in the differentiation of chromatophores^29,52–54^. In zebrafish, the distinct expression patterns of *mitfa* and *tfec* are likely to define the three chromatophore lineages: *mitfa* in melanophores, *tfec* in iridophores and both in xanthophores^18,55^. In corn snakes, *MITF* is expressed by all chromatophores, whereas *TFEC* is exclusively expressed by iridophores. Bearded dragon iridophores share the exclusive expression of *TFEC*, but they do not express at all *MITF* contrary to their corn snake counterparts (Fig. 6b).

In Squamates, we currently lack the functional experiments needed to verify the exact role of these elements, and we mainly base ourselves on spontaneous mutants to deduce it. We have previously shown that Texas rat snake *MITF* mutants are leucistic, lacking both melanophores and xanthophores^51^. In ball pythons—which lack iridophores—*TFEC* mutants have reduced numbers of melanophores and xanthophores, resulting in a piebald phenotype^19,53^. In the anole lizard, gene-edited *TFEC* mutants lose their iridophores^53^. *PAX7* leopard geckos mutants lack both xanthophores and iridophores^28^. The above observations suggest a plasticity in the function of *MITF* and *PAX7* across distinct evolutionary lineages, whereas *TFEC* seems to be consistently linked to the differentiation of iridophores (Fig. 6c).

### Comparative integration of corn snake and bearded dragon chromatophores

**Fig. 7:**
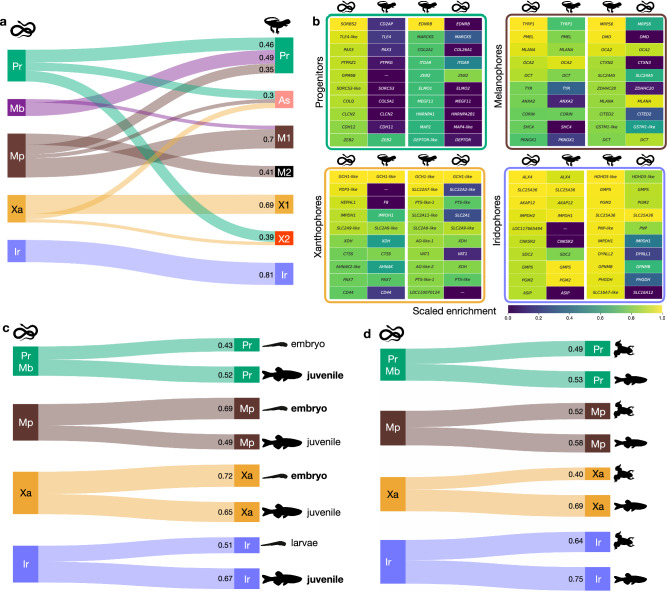
Cross-phyla integration of corn snake and zebrafish chromatophores. **a** Sankey plot summarising correspondence between corn snake and bearded dragon chromatophore clusters. Only cluster alignments with scores ≥ 0.1 are shown; edges are scaled proportionally to alignment scores. Edges with scores ≥ 0.3 are annotated with the corresponding value. **b** Comparison of differentially expressed (DE) genes in the chromatophore clusters of each species. For each cell type, we provide the top DE genes in the corn snake and their homologues in the bearded dragon, and vice versa. The heatmaps represent scaled enrichment measured by SAMap. Enrichment was assessed using a two-sided Wilcoxon rank-sum test. Z-scores were normalised per cluster to a maximum of 1; negative values were set to zero. **c** Sankey plot summarising the correspondence between corn snake and zebrafish chromatophore clusters. Stages with highest scores are highlighted in bold. **d** Sankey plot summarising the correspondence between corn snake, zebrafish, and African clawed frog chromatophore clusters. In zebrafish, the metamorphic/juvenile datapoint corresponds to the annotated chromatophore clusters in the merged 7.2 and 11 SSL samples from Saunders et al., 2019. For the African clawed frog, the datapoints correspond to the chromatophore clusters identified in merged tadpole and adult skin stages in Liao et al., 2022. Pr-MB merged progenitors–melanoblasts, Mp melanophores, Xa xanthophores, Ir iridophores.

Corn snakes and bearded dragons diverged 161 million years ago (MYA), and as our analyses show, although the differentiation of their main chromatophore lineages shares important commonalities, it also exhibits notable differences that we sought to identify through the integration of the single-cell datasets from both species. We integrated the two datasets using SAMap^52^. Briefly, SAMap integrates single-cell transcriptomes by (i) constructing a gene-gene correspondence graph using reciprocal BLASTP and including many-to-many relationships, (ii) projecting datasets into a shared manifold, and (iii) iteratively refining cross-species gene relationships to identify similar cell types (Supplementary Fig. 8a). Cluster alignment scores are computed based on the average number of mutual nearest cross-species neighbours for each cell, relative to the maximum possible number of neighbours (Fig. 7a).

Corn snake progenitor cells had a similar expression profile to subsets of bearded dragon progenitors, including *ASIP+* cells and the secondary xanthophore population. These connections further support the undifferentiated state of the latter two cell types, as proposed previously. Corn snake melanoblasts mostly corresponded with bearded dragon progenitor cells, consistent with the absence of an equivalent cluster in the bearded dragon dataset. Most melanophores matched with melanophores 1 and 2. Alignment scores were greatest between corn snake xanthophores and xanthophores 1, with lower scores connecting them with the *ASIP*+ cluster and xanthophores 2. These scores reflect the commonalities in their transcription profile already discussed. The iridophore clusters of the two species exhibited the greatest alignment score, followed by the primary melanophores and xanthophores.

To further investigate these correspondences, we selected the top ten upregulated genes in each chromatophore cluster and computed the enrichment of their SAMap-predicted orthologs (Fig. 7b, Supplementary Fig. 9). Overall, mature chromatophores upregulate similar genes, reflecting their shared function in pigment production, whereas progenitor clusters display distinct transcriptomic profiles. In fact, the corn snake progenitor population shares similar transcriptional profiles with three bearded dragon progenitor populations, each associated with a distinct role (Fig. 7a).

### Cross-phyla integration of corn snake and zebrafish chromatophores

To assess conservation of chromatophore gene expression profiles across deep evolutionary timescales, we integrated the corn snake dataset with single-cell sequencing data from zebrafish that diverged 429 MYA. The zebrafish undergoes metamorphosis, so we selected datasets spanning the entire chromatophore development, including embryonic samples (10–24 hours post-fertilisation [hpf])^56^, larval stages (10–120 hpf)^51^, metamorphic (7.2 standard somite length [SSL], ~14 dpf), and juvenile (11 SSL, ~30–40 dpf)^17^ (Fig. 7c and Supplementary Figs. 8b, 10a, 11). For the zebrafish, the metamorphic and juvenile datapoints were merged, as were the corn snake progenitor and melanoblast populations.

All zebrafish progenitor populations showed low levels of alignment score with the corn snake progenitors, besides a slightly higher value for the juvenile cluster (Fig. 7c). Indeed, comparison of the differentially expressed markers reflected little overlap in the transcriptional profile of these cells (Supplementary Fig. 11). Alignment scores were higher among the melanophore clusters, particularly the embryonic one. Similar to the corn snake and bearded dragon dataset integration, mainly the upregulation of melanogenesis components contributed to this alignment. Larvae zebrafish xanthophores contain pteridines, whereas the adult xanthophores rely on carotenoids for the bright yellow coloration^17,57^. Corn snake pterinosomes contain concentric lamellae, and their red coloration is likely due to pteridines. Consistent with these observations, corn snake xanthophores show a greater affinity to embryonic zebrafish xanthophores than juvenile ones, both strongly expressing a *GCH* paralog (Supplementary Fig. 11). At the metamorphic stage, zebrafish xanthophores are mostly cryptic, and their transcriptomic profile is therefore less similar to that of pigmented corn snake xanthophores. Iridophores differentiate later in zebrafish larvae, compared to melanophores and xanthophores^18,58^. Indeed, we observe higher alignment scores at the larvae compared to the embryonic stage and the greatest correspondence with corn snake iridophores is at the juvenile stage. Both populations highly express *ALX4* and *AKAP12*. While *AKAP12*’s function in iridophore biology remains largely unexplored in zebrafish, studies suggest its potential role in reflectance in the toad-headed lizard^59^. In zebrafish, *AKAP12* knock-outs present a severe muscle impairment and probably do not reach adulthood^60^.

Next, we simultaneously integrated the corn snake chromatophores dataset with datasets from zebrafish metamorphic/juvenile and amphibian chromatophores (Fig. 7d and Supplementary Figs. 8c, 10b, 12). The African clawed frog (*Xenopus laevis*) is a metamorphic species that diverged 351 MYA, and a dataset is available combining tadpole and adult chromatophores^61^. Overall, alignment scores were greater between the corn snake and zebrafish clusters rather than with the frog ones. Differences were smaller for melanophores and progenitors, followed by iridophores and greater for xanthophores. These differences probably reflect the different stages of maturation of the cells compared. A more comprehensive *Xenopus laevis* dataset with a stronger focus on chromatophores would be necessary to fully evaluate the divergence of these cell types.

## Discussion

Most information on reptilian coloration comes from external observations and histological analyses that focus on the presence, distribution and morphology of the three chromatophore types in the skin^12,62,63^. However, recent studies on the genetic determination of the diverse phenotypes encountered within snake and lizard emerging model species shed light on the developmental mechanisms involved in the establishment of these skin coloration patterns^5,10,13,19,28,53,54,64^. Here, we analysed the transcriptomic profile of differentiating chromatophores in two squamate species, the corn snake and the bearded dragon, by sampling multiple developmental stages. In the corn snake, we identified the three canonical chromatophore types, expressing markers previously established in the zebrafish^17,48^, and we characterised their differentiation trajectories.

In the bearded dragon, we characterised secondary populations of melanophores and xanthophores expressing a combination of progenitor and mature chromatophore markers. Their shared expression profile places them closer together in our cluster correlation analyses and strongly suggests that these are distinct cell types rather than precursors of melanophores and xanthophores. With WISH experiments, we show that these cells form lateral bands, likely contributing to the pattern formation. The presence of an *ASIP*+ cell population on the ventral skin of the embryos suggests that the Agouti pathway contributes to the ventralisation of the skin, as is the case in the zebrafish and the mouse^45,47^. Yet, in the corn snake, we observe an iridophore-specific *ASIP* expression both dorsally and ventrally. As our analyses focus on the embryonic skin, we cannot exclude the possibility that certain chromatophore progenitors follow alternative migration paths within the embryo.

Our extensive datasets allowed us to investigate the expression profile of transcription factors known to be involved in the differentiation of chromatophores. *MITF* shows a widespread expression in both species, whereas it is primarily expressed by melanophores in the zebrafish. *TFEC* has an iridophore-specific expression in the three lineages. *PAX7* is highly expressed in xanthophores and, depending on the species, in iridophores as well. As previously demonstrated in zebrafish, expression profiles are not sufficient to ascertain the implication of a transcription factor in the differentiation of a cell type. Their physical interactions, like the one between Mitfa and Tfec, can also influence the development of chromatophores^55^. Functional analyses will be necessary to elucidate the role of these transcription factors in the differentiation of reptilian chromatophores.

Integration of corn snake and bearded dragon embryonic skin datasets, as well as comparisons at larger evolutionary scales with two metamorphic species, the zebrafish and the African clawed frog, shows that (i) mature chromatophores of the compared species share similar expression profiles across species due to their common function, and (ii) the progenitor cells of each lineage have different transcriptional signatures. That is, even though mature chromatophores have similar transcriptional profiles, their progenitors engage in different developmental trajectories to reach a similar maturation state; in this case, a cell producing pigmental or structural coloration. This indicates that evolutionary diversification primarily affects progenitor programs, whereas terminal differentiation remains constrained by functional requirements. Moreover, the presence of previously undescribed chromatophore subtypes in the bearded dragon illustrates how lineage-specific innovations can arise, expanding cellular diversity. Together, these findings show that chromatophore development provides a powerful model to understand how conserved differentiation mechanisms can be modulated to generate evolutionary novelty, a principle likely shared across many vertebrate cell types beyond pigmentation.

## Methods

### Experimentation model

This research complies with all relevant ethical regulations. Corn snakes (*Pantherophis guttatus*) and central bearded dragons (*Pogona vitticeps*) were housed and bred at the LANE animal facility, running under Geneva veterinary cantonal permit no. 1008. Sampling and imaging were performed under the experimentation permits GE24/33145 and GE366/36450 approved by the Geneva veterinary cantonal authority.

### Tissue dissociation

We sampled the dorsal skin of corn snake embryos at intervals spanning embryonic days 20 to 52 and of bearded dragon embryos from E20 to E54 (Supplementary Fig. 1). We have previously shown that at E25 the coloration pattern is already established in corn snakes^13^. At this stage, *PMEL*-expressing cells already form blotches on the dorsal skin, although melanin production begins only at E33 and the melanophore distribution becomes visible at E38. Iridophores and xanthophores become noticeable at E48. At the final sampled stage (E52), embryos closely resemble hatchlings, with only pigmentation intensity remaining to change. Comparable developmental stages were sampled for the bearded dragon. A key difference between the two species is that melanophores near the dorsal midline in the bearded dragon begin producing melanin at E26, even as the pattern is still being established. For this reason, and to obtain less differentiated progenitors, we also sampled E20 skin containing unpigmented melanophores in both species. Additional intermediate time points between the stages listed above were included to enable continuous monitoring of chromatophore differentiation.

Embryos were extracted from the eggs, rinsed in ice-cold PBS, and euthanized by decapitation. The dorsal skin was dissected from either individual or pooled embryos, as detailed in Supplementary Data 3. Tissues were collected in ice-cold Hank’s Balanced Salt Solution (HBSS; Sigma, M4780). Late-stage samples (harvested between E47 and E54) were incubated overnight at 4 °C in DMEM (ThermoFisher, 11960044) supplemented with 0.25% Dispase II (Sigma, D4693) and 10 mM HEPES pH 8 (Fisher, BP310) prior to enzymatic digestion. All tissues were then incubated in 1 mL of digestion solution containing 2.5 mg/mL Collagenase Type II (Worthington, LS004176), 2 mM CaCl₂ (Sigma, 21110), and 1U/μl DNase I (Worthington, LS002139) in HBSS. For bearded dragon samples older than E33, the digestion solution was further supplemented with 1U/μl Hyaluronidase (Worthington, LS002592). Samples were digested on a rotating wheel at 35 °C for 35 minutes. Following digestion, samples were diluted in 4 mL of HBSS containing 0.25% BSA (Sigma, A3294), centrifuged at 300 × *g* for 10 minutes, and the resulting cell pellets were resuspended in 5 mL of 0.25% BSA in HBSS. The suspension was filtered through 40μm filters (Corning, 352340), centrifuged again at 300 × *g* for 10 minutes, and resuspended in 110 μl of 0.25% BSA in HBSS. A 10 μl aliquot was taken for microscopy, and the remaining cell suspension was cryopreserved at –80 °C in a freezing medium consisting of DMEM (ThermoFisher, 11960044) with 10% foetal calf serum (Sigma, F2442) and 10% DMSO (Sigma, D2653) using a controlled-rate cooling box.

### Single-cell library construction and sequencing

Thawed cell suspensions were counted, and single-cell RNA-seq libraries were generated using the 10x Genomics Chromium Single Cell 3′ v3 kit. Libraries were sequenced on an Illumina NovaSeq 6000 platform, targeting 5000 cells and 50,000 reads per cell, resulting in approximately 250 million reads per sample. Sequencing metrics for each sample are summarised in Supplementary Fig. 2.

### scRNA-Seq data processing

Sequencing reads were aligned using the Cell Ranger count command (v7.0.0) with default parameters. For the corn snake, reads were mapped to the Columbia University chromosome-length genome assembly (NCBI accession GCF_029531705.1)^65^, and to its mitochondrial genome (NCBI accession AM236349.1). Counts mapped to misannotated *PAX7*-like transcript *LOC132709890* were discarded. For the bearded dragon, reads were aligned to the GCA_900067755.1 assembly^66^ and the corresponding mitochondrial genome (NCBI accession AB166795.1). Cell Ranger–filtered gene expression matrices from all stages were merged and processed using scanpy (v1.9.5). Genes detected in fewer than three cells were excluded. Cells were retained only if they expressed at least 800 genes, had less than 30% mitochondrial gene content, and showed a doublet score below 0.15 as calculated by Scrublet v0.2.3^67^. Gene expression data were regressed against cell cycle scores and mitochondrial gene content using the scanpy.pp.regress_out function in scanpy. Expression values were normalised to a uniform depth of 10,000 read counts per cell using the scanpy.pp.normalize_per_cell function and subsequently underwent a logarithmic transformation using the scanpy.pp.log1p function. The data was scaled using scanpy.pp.scale. Principal component analysis (PCA) was performed using scanpy.tl.pca with default parameters. Batch correction was performed using scanpy.external.pp.bbknn, with developmental time samples specified as the batch key and 40 principal components (n_pcs=40) retained. UMAP embeddings were generated with scanpy.tl.umap, and clusters were identified with the Leiden algorithm (scanpy.tl.leiden) at multiple resolutions to refine granularity. Cluster identities were subsequently curated by integrating Leiden-based clustering with expression of established marker genes to assign biologically meaningful cell-type labels. The chromatophore cluster was subsetted and reclustered using the same Leiden clustering approach. Differential gene expression analysis was performed with sc.tl.rank_genes_groups using the Wilcoxon rank-sum test (method = ‘wilcoxon’).

Cell types were identified based on the expression of established markers (Supplementary Fig. 3a). Two large fibroblast clusters marked by robust *COL1A1* expression represented 84.5% and 71.8% of total cells in the corn snake and the bearded dragon datasets, respectively. In both species, these clusters distinctly expressed markers of the upper and lower dermis^68^ (Supplementary Fig. 3b). The remaining clusters correspond to epidermal cells, including keratinocytes, marked by *DSP* expression, vascular endothelial cells expressing *TIE1*, erythrocytes characterized by *HBB-b1* expression, and immune cells marked by *PTPRC*. Skeletal and smooth muscle cells were identified by the expression of *MYOD1* and *ACTA2*, respectively.

### Connectivity graphs, trajectory analyses and gene expression cascades in chromatophores

The PAGA graphs were generated using scanpy.tl.paga with default parameters. URD models differentiation by simulating diffusion through gene expression space, assigning cells a pseudotime, and tracing lineage relationships using biased random walks. Filtered and annotated raw count matrices, containing only high-quality chromatophore cells that passed quality control thresholds, were used as input for the trajectory trees computation with URD version 1.1.1^34^, as described in the tutorial^69^. Diffusion maps were computed using the calcDM function with parameters set to knn = 100 and sigma = 25 for the corn snake, and knn = 200 and sigma = 16 for the bearded dragon. Pseudotime was computed in URD using the flood simulation approach. Root cells were defined as the 20 E20-stage progenitor cells nearest to the centroid of the progenitor cluster in UMAP space. This centroid was computed as the mean UMAP position, and Euclidean distances were used to identify the nearest cells, representing the most transcriptionally central population at this stage. Tree tips were defined as terminal-stage cells: for the corn snake, E52 cells within the xanthophore, melanophore, and iridophore clusters; for the bearded dragon, E54 cells within the *ASIP+* population, melanophore populations 1 and 2, xanthophore populations 1 and 2, and the iridophore cluster. Flood simulations were performed with 50 simulations (*n* = 50) and a minimum of 2 cells flooded per simulation (minimum.cells.flooded = 2) using the function floodPseudotime. The resulting simulations were then consolidated into a final pseudotime ordering with floodPseudotimeProcess. Developmental trees were constructed from pseudotime-biased random walks between root and terminal populations using the buildTree function in URD, applying the preference divergence method with 25 cells per pseudotime bin, 8 bins per window, and a significance threshold of *p* < 0.001. To visualise lineage topology in three dimensions, a force-directed layout was computed from robustly visited cells (*visitfreq.raw* ≥ 0.5) using the treeForceDirectedLayout function with the Fruchterman–Reingold algorithm (*num.nn* = 600, *cut.unconnected.segments* = 10, *min.final.neighbours* = 4). Chromatophore types were assigned distinct colours, and the layout was rendered and continuously rotated with the rgl package (movie3d, *rpm* = 5) to generate the animated lineage tree.

We selected the top 50 differentially expressed genes for each trajectory based on their AUCPR.all values as computed by the aucprTestAlongTree function. Gene expression along the corresponding pseudotime trajectories was quantified using geneCascadeProcess with the following parameters: moving.window = 3, cells.per.window = 30, and scale.data = TRUE. The resulting scaled smooth expression matrices were hierarchically clustered and visualised using the seaborn.heatmap function. We then applied URD’s gene cascade tool to analyse the dynamic expression patterns of specific markers along each developmental trajectory. This approach identifies lineage-specific genes and models their expression dynamics using an impulse function to determine their onset and offset timing.

### Cross-species data integration with SAMap

The datasets used for cross-species integration consisted of raw count matrices and corresponding cell-type annotations obtained from publicly available datasets published by Wagner et al. (2018)^56^, Saunders et al. (2019)^17^ and Sur et al. (2023)^51^ for zebrafish and Liao et al. (2022)^61^ for the frog. Prior to data integration with SAMap (v1.0.15), we generated correlation maps by comparing coding sequences (CDS) across datasets using the map_genes.sh script provided on the SAMap GitHub repository. Specifically, we used zebrafish Ensembl release 91 (GRCz10) for the Wagner dataset, zebrafish Ensembl release 93 (GRCz11) for the Saunders dataset, zebrafish Ensembl release 99 (GRCz11) for the Sur dataset and Xenopus_laevis_v9.1 for the Liao dataset. The CDS files for our corn snake and bearded dragon samples were obtained from the genome assemblies referenced in the scRNA-seq data processing section. For each gene, only the longest CDS was retained for analysis. Processed single-cell expression matrices (raw counts with metadata) were loaded as.h5ad objects. SAMap objects were initialised with these datasets and corresponding gene homology maps, and integration was performed in pairwise mode to construct a shared manifold embedding across species as described in the SAMap tutorial. This approach iteratively aligns cells based on both gene expression similarity and inferred orthology, enabling identification of transcriptionally analogous cell states between species^70^. For the corn snake-zebrafish integration, each zebrafish dataset was integrated as a separate input file. Cluster alignment scores were calculated using the get_mapping_scores function with default parameters. Marker gene enrichment was assessed with the Wilcoxon rank-sum test. Resulting *z*-scores represent gene enrichment within each cluster. These scores were normalised within each species by dividing by the maximum absolute *z*-score per cluster, and negative values were set to zero to highlight positively enriched genes. For the corn snake-bearded dragon integration, cluster alignment scores were visualised using a Sankey plot generated with SankeyMATIC (sankeymatic.com). For the corn snake-zebrafish and the corn snake-zebrafish-Xenopus integrations, corn snake progenitor and melanoblast clusters were merged into a single cluster to facilitate visualisation and support meaningful biological interpretation. Cluster alignment scores (Supplementary Data 4) were represented using heatmaps generated with the seaborn.heatmap function.

### Orthologs expression heatmaps

To assess gene expression enrichment across chromatophore lineages, we used Scanpy to identify the top 10 marker genes per cluster based on enrichment scores (score field in sc.tl.rank_genes_groups, method=wilcoxon). Gene orthology between species was established using the SAMap gene-pair querying framework. For each top marker gene in the query clusters, putative orthologs in the compared species were identified using transcript-level matches derived from SAMap. SAMap can return up to two orthologs per gene: one based on transcriptomic correlation, and one based on BLAST sequence similarity. These orthologs may correspond to the same gene or to distinct candidates. In some cases, no ortholog is identified by either method. When both BLAST-based sequence similarity and SAMap-derived transcriptomic correlation scores were available, the ortholog associated with the lower *p*-value was selected, prioritising the most statistically supported match. If one of the *p*-values is unavailable, the SAMap correlation-based match is used as a fallback. If no correlation-based match is available, the BLAST-based ortholog is selected instead. For heatmap visualisation, scanpy enrichment scores were normalised per cluster to a maximum value of 1 and clipped at 0 to focus on positively enriched features. All ortholog correlation *p*-values are provided in Supplementary Data 5.

### Imaging

Staged embryos were fixed in 4% paraformaldehyde, washed in progressively increasing concentrations of MetOH and imaged using a VHX-6000 digital microscope (Keyence). Cell suspensions in 0.25% BSA HBSS obtained from tissue dissociation were imaged using a VHX-7000 digital microscope (Keyence).

### Whole-mount in situ hybridisations

We designed species-specific digoxigenin-labelled antisense riboprobes for corn snake *ASIP* and bearded dragon *ASIP*, *ADGRG1* and *PMEL* (Supplementary Table 1). Embryos at different developmental stages were fixed in 4% paraformaldehyde and dehydrated in methanol. Whole-mount in situ hybridisations (WISH) were performed as previously described^71^. Bearded dragon embryos were staged based on *Anolis* staging^72^. Note that on the same embryonic day, embryos can be at a different developmental stage. Corn snake embryos were staged as previously described^13^. Embryos were imaged using the VHX-6000 (Keyence).

### Statistics and reproducibility

No statistical method was used to predetermine sample size. Sample sizes were determined by the number of embryos available at each developmental stage and the yield of viable cells following tissue dissociation. Sex was not considered in the study design, as chromatophore differentiation is not known to be sexually dimorphic at embryonic stages in these species. No data were excluded from the analyses, besides low-quality cells based on pre-defined quality control thresholds. The experiments were not randomised. The investigators were not blinded to allocation during experiments and outcome assessment. Whole-mount in situ hybridisation experiments were performed on at least two embryos per probe with consistent results.

### Reporting summary

Further information on research design is available in the Nature Portfolio Reporting Summary linked to this article.

## Supplementary information

Supplementary Information

Description of Additional Supplementary Files

Supplementary Data 1

Supplementary Data 2

Supplementary Data 3

Supplementary Data 4

Supplementary Data 5

Supplementary Movie 1

Supplementary Movie 2

Reporting Summary

Transparent Peer Review file

## Data Availability

The raw FASTQ files and processed H5AD files are available in the Gene Expression Omnibus (GEO) under accession code GSE303854.
