# Supplementary material for: Conserved and lineage-specific mechanisms drive chromatophore differentiation in reptiles
Source: Nat Commun. 2026 Jul 6;17:8368. doi: 10.1038/s41467-026-75281-5 (PMC13473439; doi:10.1038/s41467-026-75281-5)
Supplement: Supplementary file 1 [file 41467_2026_75281_MOESM10_ESM.pdf]

nature portfolio

Corresponding author(s): Athanasia C. Tzika

Last updated by author(s): Apr 17, 2026

Reporting Summary

Nature Portfolio wishes to improve the reproducibility of the work that we publish. This form provides structure for consistency and transparency in reporting. For further information on Nature Portfolio policies, see our Editorial Policies and the Editorial Policy Checklist.

Statistics

For all statistical analyses, confirm that the following items are present in the figure legend, table legend, main text, or Methods section.

n/a	Confirmed	
☐	☒	The exact sample size (*n*) for each experimental group/condition, given as a discrete number and unit of measurement
☐	☒	A statement on whether measurements were taken from distinct samples or whether the same sample was measured repeatedly
☐	☒	The statistical test(s) used AND whether they are one- or two-sided<br>*Only common tests should be described solely by name; describe more complex techniques in the Methods section.*
☒	☐	A description of all covariates tested
☐	☒	A description of any assumptions or corrections, such as tests of normality and adjustment for multiple comparisons
☐	☒	A full description of the statistical parameters including central tendency (e.g. means) or other basic estimates (e.g. regression coefficient) AND variation (e.g. standard deviation) or associated estimates of uncertainty (e.g. confidence intervals)
☐	☒	For null hypothesis testing, the test statistic (e.g. *F*, *t*, *r*) with confidence intervals, effect sizes, degrees of freedom and *P* value noted<br>*Give P values as exact values whenever suitable.*
☒	☐	For Bayesian analysis, information on the choice of priors and Markov chain Monte Carlo settings
☒	☐	For hierarchical and complex designs, identification of the appropriate level for tests and full reporting of outcomes
☒	☐	Estimates of effect sizes (e.g. Cohen's *d*, Pearson's *r*), indicating how they were calculated

Our web collection on statistics for biologists contains articles on many of the points above.

Software and code

Policy information about availability of computer code

Data collection	no software
Data analysis	Cell Ranger count command (v7.0.0) - scanpy (v1.9.5) - Scrublet v0.2.3 - URD version 1.1.1 - SAMap (v1.0.15) - rgl package (1.3.24) - seaborn (0.13.2) - SankeyMATIC (https://sankeymatic.com/)

For manuscripts utilizing custom algorithms or software that are central to the research but not yet described in published literature, software must be made available to editors and reviewers. We strongly encourage code deposition in a community repository (e.g. GitHub). See the Nature Portfolio guidelines for submitting code & software for further information.

Data

Policy information about availability of data

All manuscripts must include a data availability statement. This statement should provide the following information, where applicable:

- Accession codes, unique identifiers, or web links for publicly available datasets
- A description of any restrictions on data availability
- For clinical datasets or third party data, please ensure that the statement adheres to our policy

The raw FASTQ files and processed H5AD files are available in the Gene Expression Omnibus (GEO) under accession code GSE303854.

Research involving human participants, their data, or biological material

Policy information about studies with human participants or human data. See also policy information about sex, gender (identity/presentation), and sexual orientation and race, ethnicity and racism.

Reporting on sex and gender	N/A
Reporting on race, ethnicity, or other socially relevant groupings	N/A
Population characteristics	N/A
Recruitment	N/A
Ethics oversight	N/A

Note that full information on the approval of the study protocol must also be provided in the manuscript.

Field-specific reporting

Please select the one below that is the best fit for your research. If you are not sure, read the appropriate sections before making your selection.

☒ Life sciences ☐ Behavioural & social sciences ☐ Ecological, evolutionary & environmental sciences

For a reference copy of the document with all sections, see nature.com/documents/nr-reporting-summary-flat.pdf

Life sciences study design

All studies must disclose on these points even when the disclosure is negative.

Sample size	No statistical method was used to predetermine sample size. Sample sizes were determined by embryo availability at each developmental stage and the cell yield following tissue dissociation. Multiple timepoints were sampled to ensure comprehensive coverage of chromatophore differentiation. No experimental groups were used. Samples were allocated by developmental stage to reconstruct chromatophore differentiation trajectories.
Data exclusions	No data was excluded.
Replication	In situ hybridisation experiments were replicated and all attempts at replication were successful.
Randomization	Randomisation was not relevant to this study. Samples were collected at defined developmental stages to capture the full course of chromatophore differentiation, with no group allocation or treatment conditions.
Blinding	Blinding was not relevant to this study. Experiments involved sampling embryonic skin at defined developmental stages for single-cell transcriptomic profiling, with no group allocation or treatment conditions.

Reporting for specific materials, systems and methods

We require information from authors about some types of materials, experimental systems and methods used in many studies. Here, indicate whether each material, system or method listed is relevant to your study. If you are not sure if a list item applies to your research, read the appropriate section before selecting a response.

Materials & experimental systems

n/a	Involved in the study	
☒	☐	Antibodies
☒	☐	Eukaryotic cell lines
☒	☐	Palaeontology and archaeology
☐	☒	Animals and other organisms
☒	☐	Clinical data
☒	☐	Dual use research of concern
☒	☐	Plants

Methods

n/a	Involved in the study	
☒	☐	ChIP-seq
☒	☐	Flow cytometry
☒	☐	MRI-based neuroimaging

Animals and other research organisms

Policy information about studies involving animals; ARRIVE guidelines recommended for reporting animal research, and Sex and Gender in Research

Laboratory animals	wild-type corn snake (Pantherophis guttatus) embryos and wild-type bearded dragon (Pogona vitticeps) embryos
Wild animals	N/A
Reporting on sex	N/A
Field-collected samples	N/A
Ethics oversight	Corn snakes (Pantherophis guttatus) and central bearded dragons (Pogona vitticeps) were housed and bred at the LANE animal facility running under veterinary cantonal permit no. 1008. Sampling and imaging were performed under the experimentation permits GE24/33145 and GE366/36450.

Note that full information on the approval of the study protocol must also be provided in the manuscript.

Plants

Seed stocks	N/A
Novel plant genotypes	N/A
Authentication	N/A
