# Supplementary material for: Conserved and lineage-specific mechanisms drive chromatophore differentiation in reptiles
Source: Nat Commun. 2026 Jul 6;17:8368. doi: 10.1038/s41467-026-75281-5 (PMC13473439; doi:10.1038/s41467-026-75281-5)
Supplement: Supplementary file 2 [file 41467_2026_75281_MOESM11_ESM.pdf]

nature portfolio

Peer Review File

Conserved and lineage-specific mechanisms drive chromatophore differentiation in reptiles

Corresponding Author: Dr Athanasia Tzika

This file contains all reviewer reports in order by version, followed by all author rebuttals in order by version.

Version 0:

Reviewer comments:

Reviewer #1

(Remarks to the Author)
In this manuscript Helleboid and Tzika undertake a comparative transcriptomic analysis of pigment cell lineage development, starting with their own data from multiple stages of corn snake and bearded dragon, and then integrating these findings with prior work on zebrafish and frog. The work comprises mostly results of single cell RNA-seq and dataset integrations but also includes in situ hybridization for especially informative transcripts. Overall, the authors uncover a number of interesting similarities and differences within and between phyla and the work provides an important contribution to understanding both development and evolution of these chromatophores. The computational analyses are rigorous and comprehensive, the inferences are reasonable, and the writing and figures are clear. Datasets such as these invariably raise many questions about the biology that might be addressed in additional bioinformatic or organismal analyses, but I do not see any of those being necessary for publishing the work at hand. This is particularly true given the challenging nature of working with non-traditional models such as these. I expect the study will stimulate interesting future efforts in the field and in this respect and in the findings including already the paper makes an important contribution.

Reviewer #2

(Remarks to the Author)
Using the corn snake and bearded dragon as model systems, the authors employ developmental single-cell transcriptomics to address two main questions: (1) the diversity of reptilian pigment cells, their differentiation trajectories, and the respective contributions of different pigment cell types to body coloration; and (2) the conserved and divergent patterns of pigment cell differentiation across vertebrates, particularly in reptiles. With respect to the first question, the authors provide a detailed interpretation of how different pigment cell types contribute to body coloration patterns and present ample evidence to support their conclusions. This work offers an important cellular and molecular perspective on vertebrate pigment pattern formation. However, the analysis supporting the second question suffers from several methodological limitations. A major concern is that the authors aim to compare pigment cell developmental conservation and diversity between two species using single-cell atlases, but it appears that the datasets were not integrated prior to downstream clustering and UMAP-based dimension reduction. Cross-species integration is critical because, without it, batch effects, species-specific expression differences, and technical variability can lead to inconsistent clustering and low-dimensional embeddings, making direct comparison of cell types unreliable. While integrating datasets across species presents challenges, such as ortholog mapping and expression variability, it is necessary for robust identification of corresponding cell populations and for accurate inference of conserved versus species-specific developmental programs. Indeed, cross-species integration could also help clarify the definition of progenitor populations: for example, if deduced progenitor cells from both species cluster together, they are more likely to represent pigment cell progenitors with conserved gene expression; if they do not cluster together, they may instead correspond to species-specific pigment cell types.
Another concern relates to the granularity of single-cell clustering, which can strongly influence downstream interpretations. If cells in different states of the same type are erroneously assigned to separate clusters, their intrinsic transcriptional similarity may be misinterpreted as evidence of differentiation trajectories or lineage relationships. This issue is particularly relevant when defining progenitor or stem-like populations. In the present study, although the authors provide substantial evidence that the “progenitor” cluster shares transcriptional features with pigment cells and expresses certain progenitor-associated marker genes, it cannot be conclusively determined that these cells represent bona fide common progenitors. They may instead reflect a transcriptionally intermediate state. Such possibilities cannot be excluded by pseudotime analyses such as URD. Therefore, in the absence of solid functional evidence for stemness or progenitor identity,

interpretations of lineage differentiation should be presented with caution. The authors should revised related parts accordingly.
Specific comments:
Lines 31–38, 43–44: References are required.
Lines 79–80 and Supplementary Figure 3, panel A: Please provide UMAP visualizations for SOX10, FABP7, and PMEL.
Supplementary Figure 3, panel B: The fibroblast annotations for the two species appear to be reversed.
Lines 173–182 and Figure 3d–h: The spatial distribution of ADGRG1-expressing cells (melanophores 2 and xanthophores 2) is clearly illustrated. However, the manuscript does not address their spatial relationship with melanophores 1 (which could be delineated by the differential regions between OCA2 or DCT expression and ADGRG1 expression) and xanthophores 1 (characterized by GCH1-like expression). Clarifying the relative spatial arrangement of these pigment cell subpopulations across the body surface would not only improve our understanding of their potential functional specialization, but also provide an important spatial validation of the single-cell transcriptomic classifications.
Figure 4, panels b–d: Highlight the ventral region between the forelimbs with strong ASIP signal to better support the claims in Lines 219–226.
Figure 4, panels e–f: Please use arrows or other graphical markers to highlight the pigment blotches or stripes in the images, and clearly annotate them in the figure caption. This would help readers better interpret the features discussed in the main text (Lines 227–238).
Figure 2d and Figure 5a: Please clarify the meaning of the circular outlines shown in the figures by explicitly labeling them. In addition, the methods used to generate these outlines should be described. In particular, it would be helpful to explain how the boundaries between different cell clusters were defined.
Figure 6: It is recommended to provide annotations for each deduced cell type. While the conclusions in this figure are interesting and important, the cross-species comparison may be compromised if single-cell datasets from all three species (e.g., zebrafish) are not properly integrated. Observed interspecies differences may reflect true biology, but they could also arise from batch effects, especially differences in clustering. Additionally, references supporting the zebrafish-related conclusions should be cited in the figure caption.
Figure 7 and related text: Potential batch effects in cross-species cell clustering may significantly affect these results.
Lines 446–450: The method of animal euthanasia should be clearly described.
Lines 547 and 553: The coding sequence (CDS) is redundantly defined; please remove the repetition.
Line 679: The reference information is incomplete and the issue number is missing.

Reviewer #3

(Remarks to the Author)
The manuscript entitled ‘Conserved and lineage-specific mechanisms drive chromatophore differentiation in reptiles’ revealed the mechanism of chromatophore development in corn snakes and bearded dragon using scRNA-seq data and whole-mount in situ hybridizations. The discovery of novel chromatophore subtypes and the comparative evolutionary framework are particularly compelling. This is an interesting and comprehensive study that makes significant contributions to the fields of evolutionary developmental biology and pigment cell biology. However, in its current form, the authors need to address the concerns below, which primarily aim to strengthen the interpretation and discussion of the findings:
1. The discovery of “secondary chromatophores” is interesting, but their functions remain speculative. The hypothesis that they are “unpigmented or eventually acquire a different shade” (Lines 183-184) needs further support, especially its validated functions in the final skin coloration pattern.
2. The proposed mechanism involving ADGRG1, collagen III, and TGM2 (Lines 185-192) is interesting but highly preliminary. This section should be tempered or possibly moved to the discussion.
3. For the bearded dragon secondary chromatophores, are there any similar cell states in the zebrafish or frog datasets (e.g., RUNX3-expressing pigment cells)?
4. The term “secondary chromatophores” is potentially confusing—are they a new cell type, a transitional state, or a microenvironmentally modulated subtype?
5. ASIP, a protein commonly associated with melanin synthesis, requires further elucidation regarding its role in corn snakes, particularly concerning its relationship with iridophore differentiation. This could be expanded upon in the discussion.
6. Perhaps a SCENIC-based comparison of transcription factor regulation activity in chromatophore cells across corn snake, bearded dragon, and zebrafish would better directly visualize expression profiles across species to elucidate the convergence of regulatory programs.
7. Lines 249-250: The strong connection of melanophores 2 and xanthophores 2, and similar expression pattern of marker genes suggest whether they might be the same type of cell ?
8. Figure 2 & 5: The URD trajectory plots are visually cluttered. Consider highlighting the key rows or adding detailed gene expression trends to supplementary panels.
9. Figure 3 & 4: In all in situ hybridization results, it would be preferable for authors to highlight the concerned signals with arrows or circles.
10. Lines 271–296: The transcription factor description section is redundant. It is recommended to add a table in the appendix that compares and summarizes the expression patterns and functional information of different TFs in different species.
11. The Methods section should better include a brief justification for the chosen embryonic time points, explaining how they cover key windows of chromatophore differentiation.
12. Line 533: The criteria for screening the top 50 differentially expressed genes here are unclear. It is recommended to provide additional justification.
13. Please ensure that the manuscript formatting meets the requirements，e.g., Line 841 & 842.

Version 1:

Reviewer comments:

Reviewer #2

(Remarks to the Author)
The authors have provided detailed revisions and explanations addressing my concerns, and I have no further questions. Congratulations to the authors on this nice work.

Reviewer #3

(Remarks to the Author)
The author has effectively settled all my issues.

Open Access This Peer Review File is licensed under a Creative Commons Attribution 4.0 International License, which permits use, sharing, adaptation, distribution and reproduction in any medium or format, as long as you give appropriate credit to the original author(s) and the source, provide a link to the Creative Commons license, and indicate if changes were made.
In cases where reviewers are anonymous, credit should be given to 'Anonymous Referee' and the source.
The images or other third party material in this Peer Review File are included in the article's Creative Commons license, unless indicated otherwise in a credit line to the material. If material is not included in the article's Creative Commons license and your intended use is not permitted by statutory regulation or exceeds the permitted use, you will need to obtain permission directly from the copyright holder.
To view a copy of this license, visit https://creativecommons.org/licenses/by/4.0/

Conserved and lineage-specific mechanisms drive chromatophore differentiation in reptiles

Main revision points

Thanks to the comments of the reviewers, we could improve the clarity of the manuscript. Based on the comments of the reveiwers, we focused on two main points: the integration of the different single-cell datasets and the characterisation of the novel cell types described in the bearded dragon. The revision necessitated additional whole-mount in situ hybridisations as well as in silico analyses. The results are presented here for the appreciation of the reviewers but we chose not to include them in the main text which is already very extensive. Indeed, these analyses, such as the SCENIC regulon identification, present excellent starting points for the continuation of the present study.

We also took the opportunity to correct typos and grammatically improve the text. Following the recommendation of a neurobiologist and as defined in Solovieva & al, 2021, we also renamed the 'radial glial cells' cluster to 'Schwann cell precursors'. Below you will find our point-by-point answers to the comments of the reviewers.

Point-by-point answers

Reviewer #1:

In this manuscript Helleboid and Tzika undertake a comparative transcriptomic analysis of pigment cell lineage development, starting with their own data from multiple stages of corn snake and bearded dragon, and then integrating these findings with prior work on zebrafish and frog. The work comprises mostly results of single cell RNA-seq and dataset integrations but also includes in situ hybridization for especially informative transcripts. Overall, the authors uncover a number of interesting similarities and differences within and between phyla and the work provides an important contribution to understanding both development and evolution of these chromatophores. The computational analyses are rigorous and comprehensive, the inferences are reasonable, and the writing and figures are clear. Datasets such as these invariably raise many questions about the biology that might be addressed in additional bioinformatic or organismal analyses, but I do not see any of those being necessary for publishing the work at hand. This is particularly true given the challenging nature of working with non-traditional models such as these. I expect the study will stimulate interesting future efforts in the field and in this respect and in the findings including already the paper makes an important contribution.

We sincerely appreciate the reviewer's encouraging evaluation of our manuscript. Indeed, working with non-traditional model species is a challenge, but rewarding with unexpected discoveries.

Reviewer #2:

Using the corn snake and bearded dragon as model systems, the authors employ developmental single-cell transcriptomics to address two main questions: (1) the diversity of reptilian pigment cells, their differentiation trajectories, and the respective contributions of different pigment cell types to body coloration; and (2) the conserved and divergent patterns of pigment cell differentiation across vertebrates, particularly in reptiles. With respect to the first question, the authors provide a detailed interpretation of how different pigment cell types contribute to body coloration patterns and present ample evidence to support their conclusions. This work offers an important cellular and molecular perspective on vertebrate pigment pattern formation.

We would like to thank the reviewer for the positive assessment of our manuscript and the constructive comments that allowed us to improve it.

However, the analysis supporting the second question suffers from several methodological limitations. A major concern is that the authors aim to compare pigment cell developmental conservation and diversity between two species using single-cell atlases, but it appears that the datasets were not integrated prior to downstream clustering and UMAP-based dimension reduction. Cross-species integration is critical because, without it, batch effects, species-specific expression differences, and technical variability can lead to inconsistent clustering and low-dimensional embeddings, making direct comparison of cell types unreliable. While integrating datasets across species presents challenges, such as ortholog mapping and expression variability, it is necessary for robust identification of corresponding cell populations and for accurate inference of conserved versus species-specific developmental programs. Indeed, cross-species integration could also help clarify the definition of progenitor populations: for example, if deduced progenitor cells from both species cluster together, they are more likely to represent pigment cell progenitors with conserved gene expression; if they do not cluster together, they may instead correspond to species-specific pigment cell types.

We agree that cross-species alignment is essential for robust comparison. In our analysis, datasets were not compared using independent within-species UMAPs; instead, we performed cross-species integration using SAMap (Tarashansky et al., eLife 2021), which constructs species-specific manifolds and then stitches them into a joint graph using mutual nearest cross-species neighborhoods informed by a gene-homology graph. In SAMap, technical batch effects are addressed through manifold-level alignment rather than expression-matrix correction: species-specific neighbor graphs are constructed independently and subsequently stitched using mutual nearest cross-species neighborhoods based on homologous genes, which reduces the impact of platform- and species-specific technical variability on the integrated representation. Notably, SAMap has been demonstrated to outperform alternative cross-species atlas integration methods in a recent benchmark analysis (Song & al, 2023). Cell-type correspondence was assessed quantitatively using SAMap mapping scores between annotated clusters, and shared gene programs were extracted using GenePairFinder.

We have revised the Methods and Results sections to include additional details describing the integration procedure. To clarify our analysis, we added the UMAPs shown in the new Supplementary Figure 8, which are derived from the SAMap-integrated embedding using the SAMap clustering function.

To further validate our use of SAMap, we additionally performed orthology-restricted cross-species integrations (corn snake - bearded dragon) using three widely used integration methods: BBKNN (Polański & al, 2019), Harmony (Korsunsky & al, 2019), and Scanorama (Hie et al, 2024). For these analyses, reciprocal best-hit homologs identified from the SAMap gene homology mappings were used to construct a shared 1:1 ortholog feature space, and raw count matrices were re-integrated with species treated as batch. The corresponding UMAPs (Reviewers Figure 1) indicated that these alternative integration approaches resulted in embeddings in which cells showed a noticeable degree of species-specific separation. By comparison, the SAMap-integrated embedding displayed more pronounced cross-species co-clustering of homologous chromatophore populations (Supplementary Fig. 8).

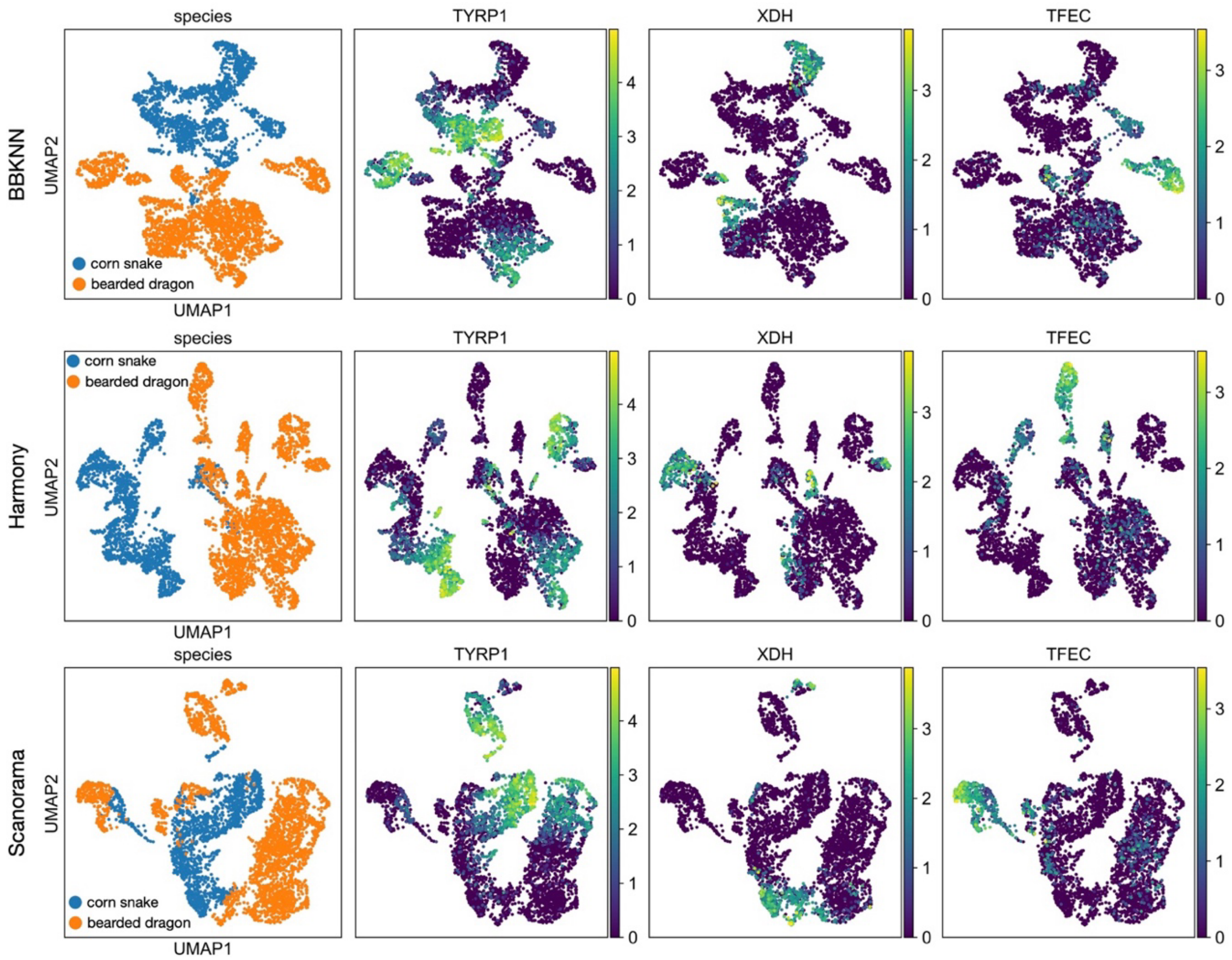

Reviewers Figure 1. Corn snake and bearded dragon chromatophore datasets were restricted to reciprocal best-hit (RBH) homologs inferred from SAMap BLAST mappings, and genes were harmonized by assigning a shared feature identifier to each 1:1 pair. Cross-species integration was then performed using three methods: BBKNN, Harmony, and Scanorama (each species treated as a batch). UMAPs show the resulting joint

embeddings colored by species. Marker UMAPs display lineage-associated genes TYRP1 (melanophores), XDH (xanthophores), and TFEC (iridophores).

References
Tarashansky & al "Mapping single-cell atlases throughout Metazoa unravels cell type evolution" (2021) eLife - https://doi.org/10.7554/eLife.66747
Song & al "Benchmarking strategies for cross-species integration of single-cell RNA sequencing data" (2023) Nature Communications - https://doi.org/10.1038/s41467-023-41855-w
Polański & al "BBKNN: fast batch alignment of single cell transcriptomes" Bioinformatics (2019) - https://doi.org/10.1093/bioinformatics/btz625
Hie & al "Scanorama: integrating large and diverse single-cell transcriptomic datasets" Nature Protocols (2024) - https://doi.org/10.1038/s41596-024-00991-3
Korsunsky & al "Fast, sensitive and accurate integration of single-cell data with Harmony" Nature Methods (2019) - https://doi.org/10.1038/s41592-019-0619-0

Another concern relates to the granularity of single-cell clustering, which can strongly influence downstream interpretations. If cells in different states of the same type are erroneously assigned to separate clusters, their intrinsic transcriptional similarity may be misinterpreted as evidence of differentiation trajectories or lineage relationships. This issue is particularly relevant when defining progenitor or stem-like populations. In the present study, although the authors provide substantial evidence that the "progenitor" cluster shares transcriptional features with pigment cells and expresses certain progenitor-associated marker genes, it cannot be conclusively determined that these cells represent bona fide common progenitors. They may instead reflect a transcriptionally intermediate state. Such possibilities cannot be excluded by pseudotime analyses such as URD. Therefore, in the absence of solid functional evidence for stemness or progenitor identity, interpretations of lineage differentiation should be presented with caution. The authors should revised related parts accordingly.

We agree with the reviewer that the progenitor clusters might include cells in differentiation. The following cautionary note has now been added in the section 'Corn snake chromatophores express established lineage markers':

"Note that the assignment to the latter cluster was based on the shared transcriptional features with pigment cells and the expression of progenitor-associated marker genes, however we cannot exclude that these cells have already engaged in differentiation, rather than being fully competent progenitors."

And in the section 'Secondary populations of melanophores and xanthophores present in the bearded dragon embryonic skin':

"As progenitors share the expression of lineage markers with the other differentiated clusters, such as a PTS paralog (PTS-like-2) with the xanthophores, MLANA and DCT with the melanophores, and GMPS with the iridophores, these cells might have actually initiated differentiation, instead of being fully competent progenitors."

Specific comments:
Lines 31–38, 43–44: References are required.

We have now added appropriate references.

Lines 79–80 and Supplementary Figure 3, panel A: Please provide UMAP visualizations for SOX10, FABP7, and PMEL.

We have added UMAPs for *SOX10* and *PMEL*. A UMAP for *FABP7* is already present as this gene is the marker for the Schwann cell precursors cluster.

Supplementary Figure 3, panel B: The fibroblast annotations for the two species appear to be reversed.

Thank you for catching this mistake. We have now reversed the UMAPs accordingly.

Lines 173–182 and Figure 3d–h: The spatial distribution of ADGRG1-expressing cells (melanophores 2 and xanthophores 2) is clearly illustrated. However, the manuscript does not address their spatial relationship with melanophores 1 (which could be delineated by the differential regions between OCA2 or DCT expression and ADGRG1 expression) and xanthophores 1 (characterized by GCH1-like expression). Clarifying the relative spatial arrangement of these pigment cell subpopulations across the body surface would not only improve our understanding of their potential functional specialization, but also provide an important spatial validation of the single-cell transcriptomic classifications.

We agree with the reviewer on the importance of the spatial distribution of these novel cell type. The relative position of melanophores 1 and melanophores 2 is illustrated in Fig. 3f, g, h. We performed whole-mount in situ hybridization (WISH) on an embryo of the appropriate developmental stage where melanophores 1 already produce melanin (Fig. 3f) using an *ADGRG1* probe (Fig. 3h). We could thus show that *ADGRG1*-expressing cells, corresponding to melanophores 2 and xanthophores 2, are located inbetween the bands of melanin-producing cells (melanophores 1). Unfortunately, we have not identified a marker for early stage xanthophore cells. As requested by the reviewer, we performed WISH with a *GCH1-like* probe but besides a few sparce dots laterally, we did not detect a clear expression pattern. Indeed, *GCH1-like* is expressed at later stages of development when WISH is not possible due to the thickness of the skin.

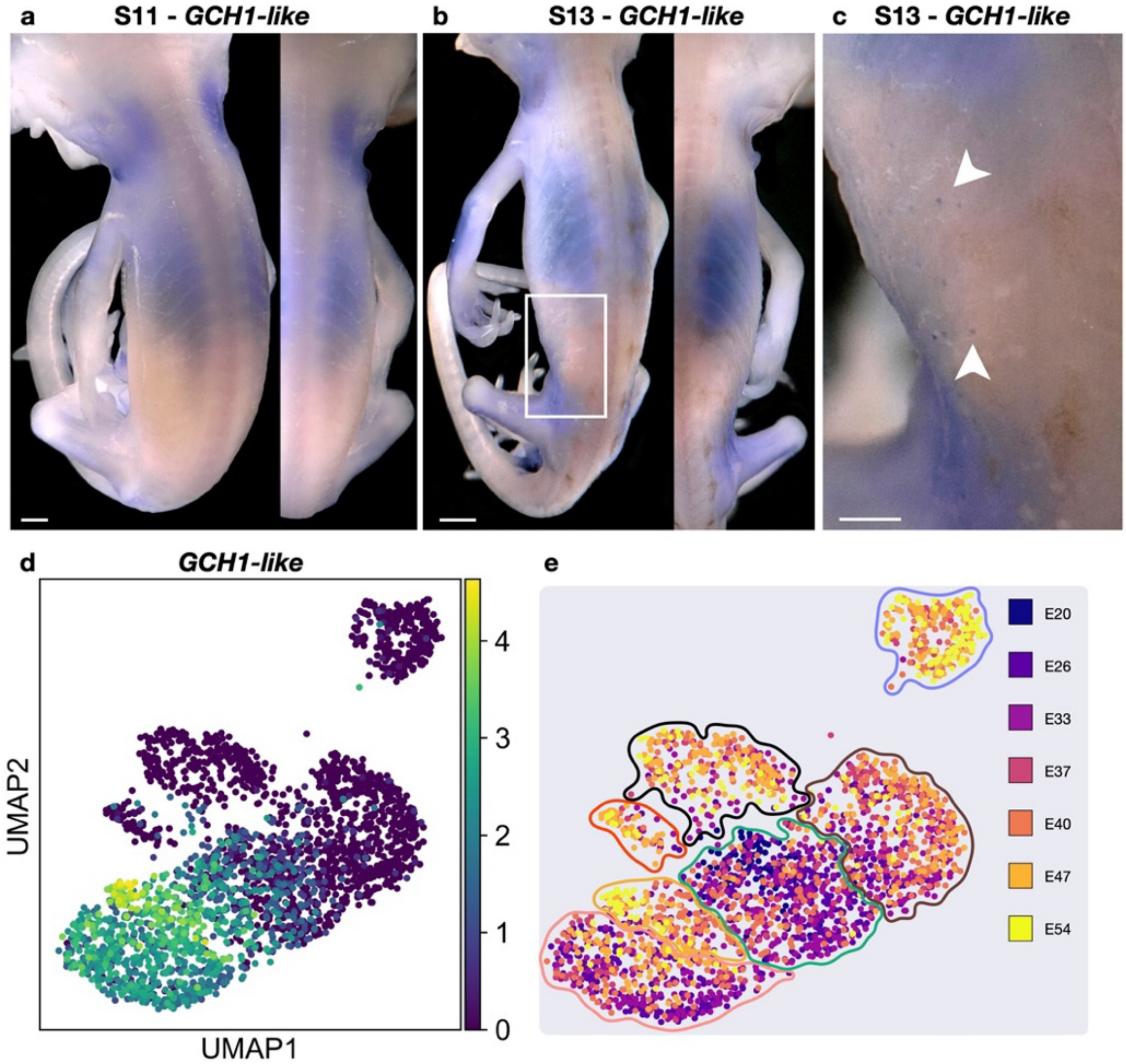

Reviewers Figure 2. a,b . Lateral (left) and dorsal (right) view of a bearded dragon embryos stained with a *GCH1-like* probe. **c.** Magnification of the highlighted area in **b**. The arrowheads point to the dots of expression. **d.** UMAP showing the expression of *GCH1-like*. **e.** UMAP of chromatophores color-coded according to the embryonic day sampled.

Figure 4, panels b–d: Highlight the ventral region between the forelimbs with strong ASIP signal to better support the claims in Lines 219–226.

An outline has been added.

Figure 4, panels e–f: Please use arrows or other graphical markers to highlight the pigment blotches or stripes in the images, and clearly annotate them in the figure caption. This would help readers better interpret the features discussed in the main text (Lines 227–238).

Arrowheads and outlines have been added.

Figure 2d and Figure 5a: Please clarify the meaning of the circular outlines shown in the figures by explicitly labeling them. In addition, the methods used to generate these outlines should be described. In particular, it would be helpful to explain how the boundaries between different cell clusters were defined.

The outlines correspond to the chromatophore cell types as shown in panel a. No additional analyses were performed. This is now explained in the figure legend.

Figure 6: It is recommended to provide annotations for each deduced cell type. While the conclusions in this figure are interesting and important, the cross-species comparison may be compromised if single-cell datasets from all three species (e.g., zebrafish) are not properly integrated. Observed interspecies differences may reflect true biology, but they could also arise from batch effects, especially differences in clustering. Additionally, references supporting the zebrafish-related conclusions should be cited in the figure caption.

Please see our answer about SAMap integration above.

Figure 7 and related text: Potential batch effects in cross-species cell clustering may significantly affect these results.

Please see our answer about SAMap integration above.

Lines 446–450: The method of animal euthanasia should be clearly described.

The information is now included in the 'Tissue dissociation' section.

Lines 547 and 553: The coding sequence (CDS) is redundantly defined; please remove the repetition.

The text was modified accordingly.

Line 679: The reference information is incomplete and the issue number is missing.

All references have been revised and completed as needed.

Reviewer #3

The manuscript entitled 'Conserved and lineage-specific mechanisms drive chromatophore differentiation in reptiles' revealed the mechanism of chromatophore development in corn snakes and bearded dragon using scRNA-seq data and whole-mount in situ hybridizations. The discovery of novel chromatophore subtypes and the comparative evolutionary framework are particularly compelling. This is an interesting and comprehensive study that makes significant contributions to the fields of evolutionary developmental biology and pigment cell biology.

We are grateful to the reviewer for their favorable assessment of our manuscript and for the valuable feedback, which has contributed to improving its clarity.

However, in its current form, the authors need to address the concerns below, which primarily aim to strengthen the interpretation and discussion of the findings:

1. The discovery of "secondary chromatophores" is interesting, but their functions remain speculative. The hypothesis that they are "unpigmented or eventually acquire a different shade" (Lines 183-184) needs further support, especially its validated functions in the final skin coloration pattern.

We agree with the reviewer that our hypothesis is not sufficiently supported by our current dataset. We thus prefer to remove this mention and instead we now state: *"Given the continuous presence of the secondary populations until the late stages of development, we assume that they participate in the final skin coloration pattern; yet their exact role remains to be elucidated. The WISH* ADGRG1 *staining suggests that they form bands between the dark bands observed in hatchlings, but we cannot confirm that these are pigment-producing cells."*

2. The proposed mechanism involving ADGRG1, collagen III, and TGM2 (Lines 185-192) is interesting but highly preliminary. This section should be tempered or possibly moved to the discussion.

In agreement with the reviewer's comment, this section has been tempered as follows:

"Therefore, we hypothesize that the migration of the secondary chromatophores could be inhibited by collagen III and their in-situ proliferation could be promoted by TGM2. However further evidence is needed to confirm this.

Given the continuous presence of the secondary populations until the late stages of development, we assume that they participate in the final skin coloration pattern; yet their exact role remains to be elucidated."

3. For the bearded dragon secondary chromatophores, are there any similar cell states in the zebrafish or frog datasets (e.g., RUNX3-expressing pigment cells)?

As the reviewer can appreciate in the Reviewers Fig. 3, we could not detect cells expressing both *Runx3* and *ADRGR1* neither by zebrafish nor by frog chromatophores.

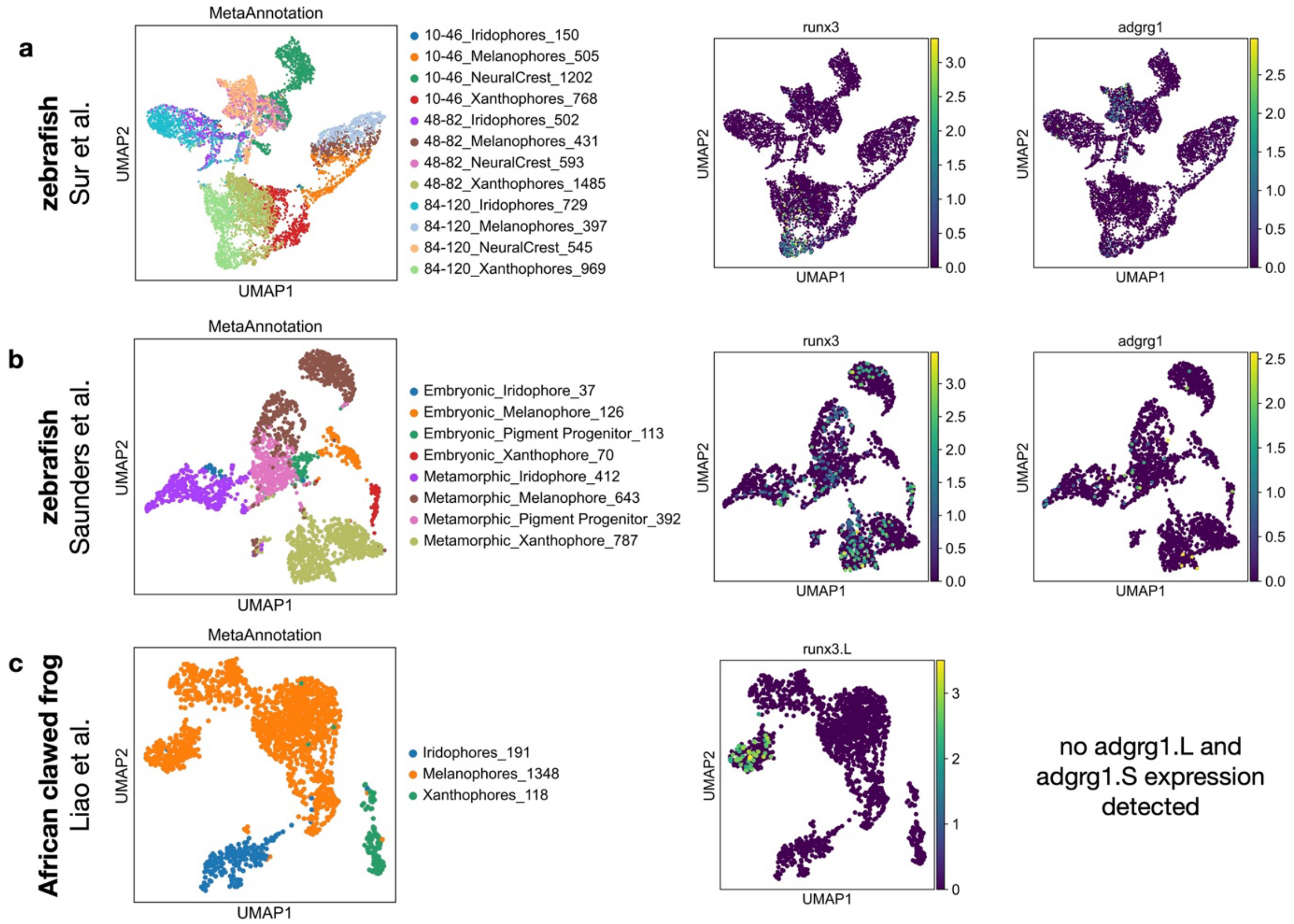

Reviewers Figure 3. Expression pattern of *runx3* and *adgrg1* in pre- and metamorphic zebrafish (**a,b**) and the African clawed frog (**c**). Chromatophore cluster annotation was retrieved from the respective publications. The number of each cell cluster is given in the annotation legend (for example, 150 cells in the '10-46 hpf iridophores'). The two frog paralogs of *adgrg1*, *adgrg1.L* and *adgrg1.S*, are not expressed in chromatophores. However, *adgrg1.L* shows expression by a small number of cells belonging to the astrocyte, oligodendrocyte, GABAergic neuron, myeloid cell, B cell, AT2 cell, cumulus cell, and erythrocyte populations, whereas *adgrg1.S* expression is not detectable in the dataset (Liao, Y. *et al.* Cell landscape of larval and adult Xenopus laevis at single-cell resolution. *Nat Commun* **13**, 4306 (2022))

4. The term "secondary chromatophores" is potentially confusing—are they a new cell type, a transitional state, or a microenvironmentally modulated subtype?

We chose the term 'secondary chromatophores' because these cells do express classical melanophore and xanthophore markers, respectively, yet the shared expression of *RUNX3* and *ADGRG1* groups them and marks them as distinct. Their strong connectivity in the PAGA graph further supports the common elements of their transcriptional profile, beyond *RUNX3* and *ADGRG1*. Secondary chromatophores differentiate simultaneous with their primary counterparts early in development and persist throughout. As we have now indicated in the main text, "*their exact role remains to be elucidated.*"

5. ASIP, a protein commonly associated with melanin synthesis, requires further elucidation regarding its role in corn snakes, particularly concerning its relationship with iridophore differentiation. This could be expanded upon in the discussion.

The role of ASIP in animal pigmentation is variable, for example shifting melanocytes from producing black pigment to yellow pigment or contributing to the ventralisation of the zebrafish coloration pattern. The spatial distribution of *ASIP*-expressing cells in the corn snake does not relate with the known distribution of iridophores in late development. Indeed, WISH was performed at stages when iridophores are not present yet and their differentiation has not initiated. Note that, in addition to iridophores and as shown in Supplementary Figure 6, ASIP is expressed by a large proportion of fibroblasts. Spatial transcriptomic analyses at different developmental stages could elucidate its role; whereas comparative analyses of the dorsal and ventral corn snake skin would be needed to identify the key players that establish such distinct patterns at each side of the animal.

These points are mentioned in the main text:

"Comparative analyses of the dorsal and ventral corn snake skin would be needed to identify the key players that establish such distinct patterns at each side of the animal, and spatial transcriptomics analyses could elucidate the role of ASIP."

6. Perhaps a SCENIC-based comparison of transcription factor regulation activity in chromatophore cells across corn snake, bearded dragon, and zebrafish would better directly visualize expression profiles across species to elucidate the convergence of regulatory programs.

As suggested by the reviewer, we performed a SCENIC-based comparison across the three species, and the results are presented in Review Figure 4. pySCENIC version 0.12.1 (Aibar et al. 2017) was used to analyse our corn snake and bearded dragon datasets using the v10 human transcription factor binding motif collection (motifs-v10nr_clust-nr.hgnc-m0.001-o0.0.tbl), and the zebrafish Saunders dataset using the zebrafish motif binding dataset described in Zeng et al. (2024). Promoters were defined as regions spanning 2,000 bp upstream and 500 bp downstream of the annotated transcription start site.

In Reviewers Fig. 4, we present the 15 most enriched regulons in progenitors, melanophores, xanthophores and iridophores compared both to all the other cell types of the datasets, as well as compared to chromatophores only. In all enriched regulon sets compared to all cell types, we find the transcription repressor BHLHE40, accompanied by BHLHE41 in the corn snake and the bearded dragon. This transcription factor is primarily linked to the circadian rhythm and to our knowledge, it has not been associated to skin coloration previously. From the well-established coloration-related transcription factors, we only detect MITF in the corn snake and the zebrafish and SOX10 in the bearded dragon, although the latter is associated with neural-crest derived cell populations in general.

Although SOX10 and MITF are undoubtably important for the differentiation of chromatophores, this analysis failed to identify established lineage-specific differentiation regulators, such PAX7 for xanthophores and ALX4 for iridophores. This limitation likely reflects two factors: (i) the reliance on a human transcription factor binding motif database for the corn snake and the bearded dragon, and (ii) the incomplete genome annotation available for non-model species.

Note that in Supplementary Table 1 the reader can already appreciate the differential expression of *BHLHE40* and *BHLHE41* by the chromatophore clusters of both species and in Supplementary Table 2 the differential expression of *MITF* by xanthophores and melanophores in both species, specifically Xanthophores 1, Melanophores 1, and Melanophores 2 for the bearded dragon.

While we believe this analysis is informative and may serve as a useful starting point for focused investigation of specific regulons, such as those associated with BHLHE40 and BHLHE41, the limitations described above currently reduce its suitability for robust assessment of global convergence in transcriptional regulatory programs across species. For this reason, we consider the analysis exploratory and potentially outside the main scope of the manuscript, unless the reviewers and editor think otherwise.

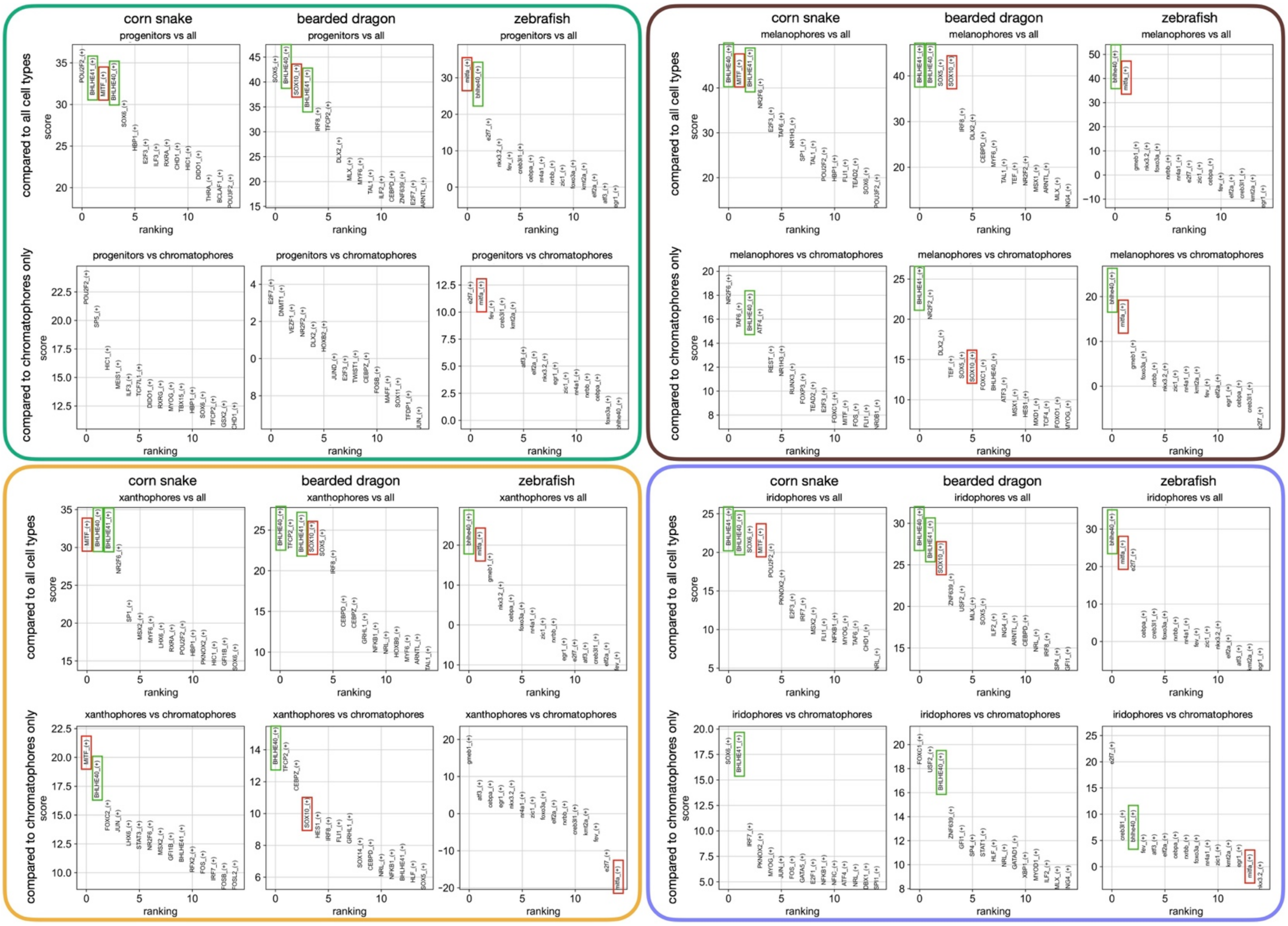

Reviewers Figure 4. Regulon activity scores inferred by SCENIC were compared using the Wilcoxon rank-sum test to identify regulons enriched in each chromatophore cluster. For each frame, in upper panels the differential regulon activity was calculated compared to all cell types in the dataset, in the lower panels, differential analysis was restricted to chromatophore populations only, using the remaining chromatophore cell types as the reference, thereby highlighting lineage-specific regulatory programs within chromatophores. The species are indicated at the top. For each lineage analyzed, chromatophore types are color-coded as follows: progenitors in green, melanophores in brown, xanthophores in yellow, and iridophores in blue.

References
Aibar et al. "SCENIC: single-cell regulatory network inference and clustering" (2017) Nature Methods - https://www.nature.com/articles/nmeth.4463
Zeng et al. "Comparative single-cell transcriptomic analysis reveals putative differentiation drivers and potential origin of vertebrate retina" (2024) NAR Genom Bioinform - https://academic.oup.com/nargab/article/6/4/lqae149/7891563

7. Lines 249-250: The strong connection of melanophores 2 and xanthophores 2, and similar expression pattern of marker genes suggest whether they might be the same type of cell?

Indeed, these two cell types share part of their transcriptional profile as attested by their strong connectivity in the PAGA graph, largely driven by the expression of *ADGRG1* and *RUNX3*. Yet they can be clearly distinguished by the expression of melanogenesis genes by Melanophores 2 (for example, *DCT* and *TYRP1*) and *PAX7* by Xanthophores 2, among others (Reviewers Fig. 5).

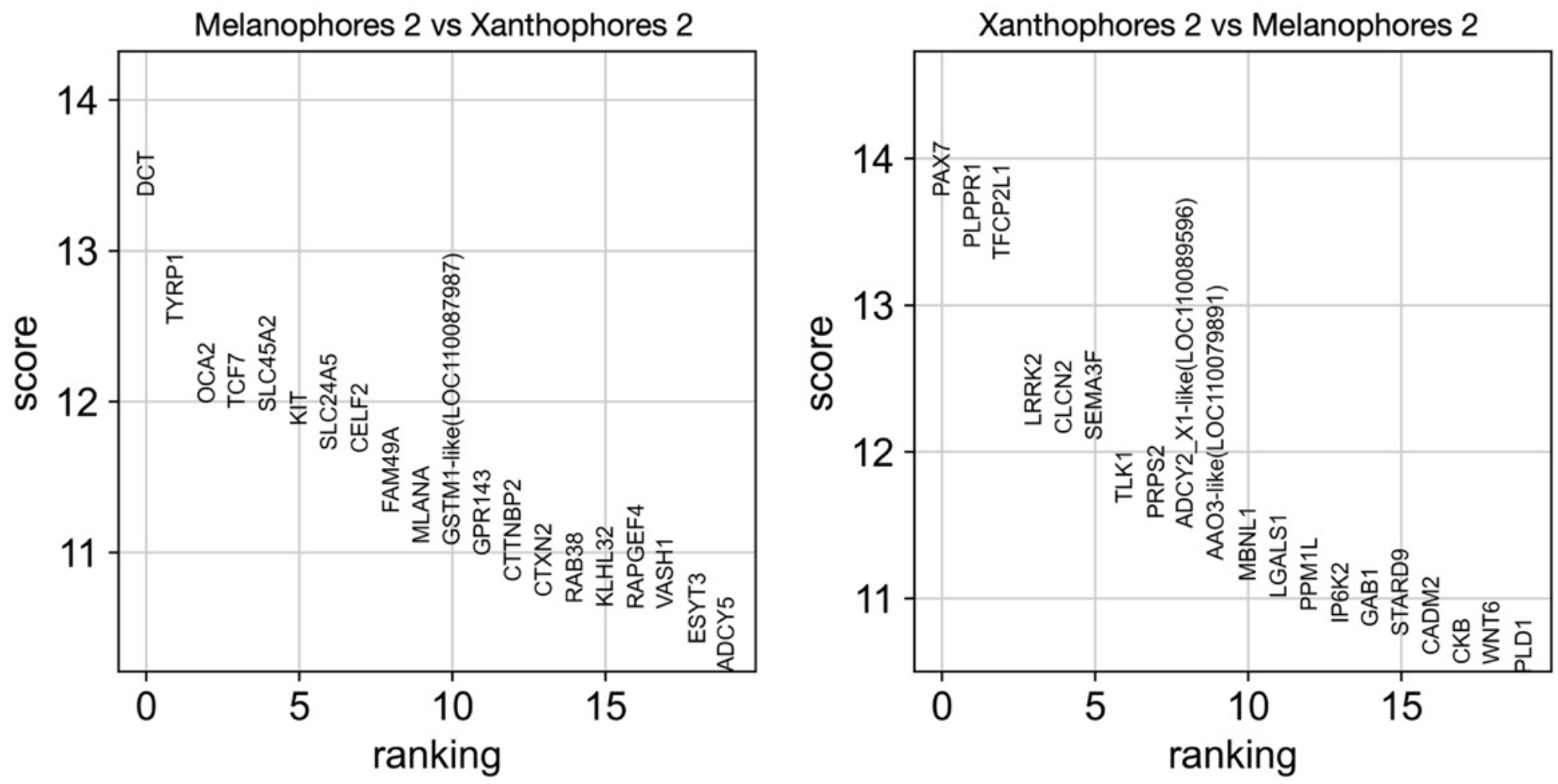

Reviewer Fig 5. Wilcoxon rank-sum test to identify the most differentially expressed genes between Melanophores 2 and Xanthophores 2.

8. Figure 2 & 5: The URD trajectory plots are visually cluttered. Consider highlighting the key rows or adding detailed gene expression trends to supplementary panels.

As suggested by the reviewer, we added frames to highlight the trajectory of the key genes discussed in the main text.

9. Figure 3 & 4: In all in situ hybridization results, it would be preferable for authors to highlight the concerned signals with arrows or circles.

We added appropriate outlines and arrowheads in both figures.

10. Lines 271–296: The transcription factor description section is redundant. It is recommended to add a table in the appendix that compares and summarizes the expression patterns and functional information of different TFs in different species.

We thank the reviewer for this comment. However, we respectfully prefer to retain the section on transcription factors in its current form. We do not believe this section is redundant within the manuscript, as it provides a focused synthesis of transcription factor roles in classical and non-classical model species and is important for contextualizing our results. For this reason, we have maintained this section without modification.

11. The Methods section should better include a brief justification for the chosen embryonic time points, explaining how they cover key windows of chromatophore differentiation.

We introduced the explanation below in the Methods:

"We sampled the dorsal skin of corn snake embryos at intervals spanning embryonic days 20 to 52 and of bearded dragon embryos from E20 to E54 (Supplementary Fig. 1). We have previously shown that at E25 the coloration pattern is already established in corn snakes. At this stage, PMEL*-expressing cells already form blotches on the dorsal skin, although melanin production begins only at E33 and the melanophore distribution becomes visible at E38. Iridophores and xanthophores become noticeable at E48. At the final stage sampled (E52), embryos closely resemble hatchlings, with only pigmentation intensity continuing to change. Comparable developmental stages were sampled for the bearded dragon. A key difference between the two species is that melanophores located near the dorsal midline in the bearded dragon begin producing melanin at E26, while the pattern is still being established. For this reason, and to obtain less differentiated progenitors, we also sampled E20 skin containing unpigmented melanophores in both species. Additional intermediate time points between the stages listed above were included to enable continuous monitoring of chromatophore differentiation."*

12. Line 533: The criteria for screening the top 50 differentially expressed genes here are unclear. It is recommended to provide additional justification.

The top 50 differentially expressed genes were selected to capture the diversity of expression patterns while retaining genes with strong cell-type specificity. Empirically, this number provided a representative overview of the major transcriptional programs underlying chromatophore differentiation without introducing redundancy from genes with highly similar expression profiles.

13. Please ensure that the manuscript formatting meets the requirements, e.g., Line 841 & 842.

All references have been completed.
