# Supplementary material for: Conserved and lineage-specific mechanisms drive chromatophore differentiation in reptiles
Source: Nat Commun. 2026 Jul 6;17:8368. doi: 10.1038/s41467-026-75281-5 (PMC13473439; doi:10.1038/s41467-026-75281-5)
Supplement: Supplementary file 3 [file 41467_2026_75281_MOESM1_ESM.pdf]

# Supplementary Information for

# Conserved and lineage-specific mechanisms drive chromatophore differentiation in reptiles

Pierre-Yves Helleboid[1] and Athanasia C. Tzika[1 *]

[1] Laboratory of Artificial & Natural Evolution (LANE), Department of Genetics & Evolution, University of Geneva, Geneva, Switzerland

***Correspondence:**
athanasia.tzika@unige.ch

## Supplementary Tables

**Supplementary table 1. Primers for the amplification of the species-specific WISH probes.**

| Species | Gene | Orientation | Name | Sequence |
|---|---|---|---|---|
| Corn snake | *ASIP* | Forward | EG_ASIPc_102F | ACCTTCTCCTGAGCTTCCTTC |
| | | Reverse | EG_ASIPc_482R | AGTTTGGATTTCCTATACGGCA |
| Bearded dragon | *ASIP* | Forward | PV_ASIPc_18F | ATATAGGAACCTTCTCCTTGGC |
| | | Reverse | PV_ASIPc_398R | TTTCCCATTCGGCAGTAGCA |
| Bearded dragon | *ADGRG1* | Forward | PV_ADGRG1c_1305F | CCACCAGGAATACCTCACCC |
| | | Reverse | PV_ADGRG1c_2065R | ACCAAAGGAAGATAAGGAAGCC |
| Bearded dragon | *PMEL* | Forward | PV_PMELc_12F | ACACAGAACCCAGGGTGAAG |
| | | Reverse | PV_PMELc_893R | CGGTAAGGTAGGTGTGGGTC |

## Supplementary Figures

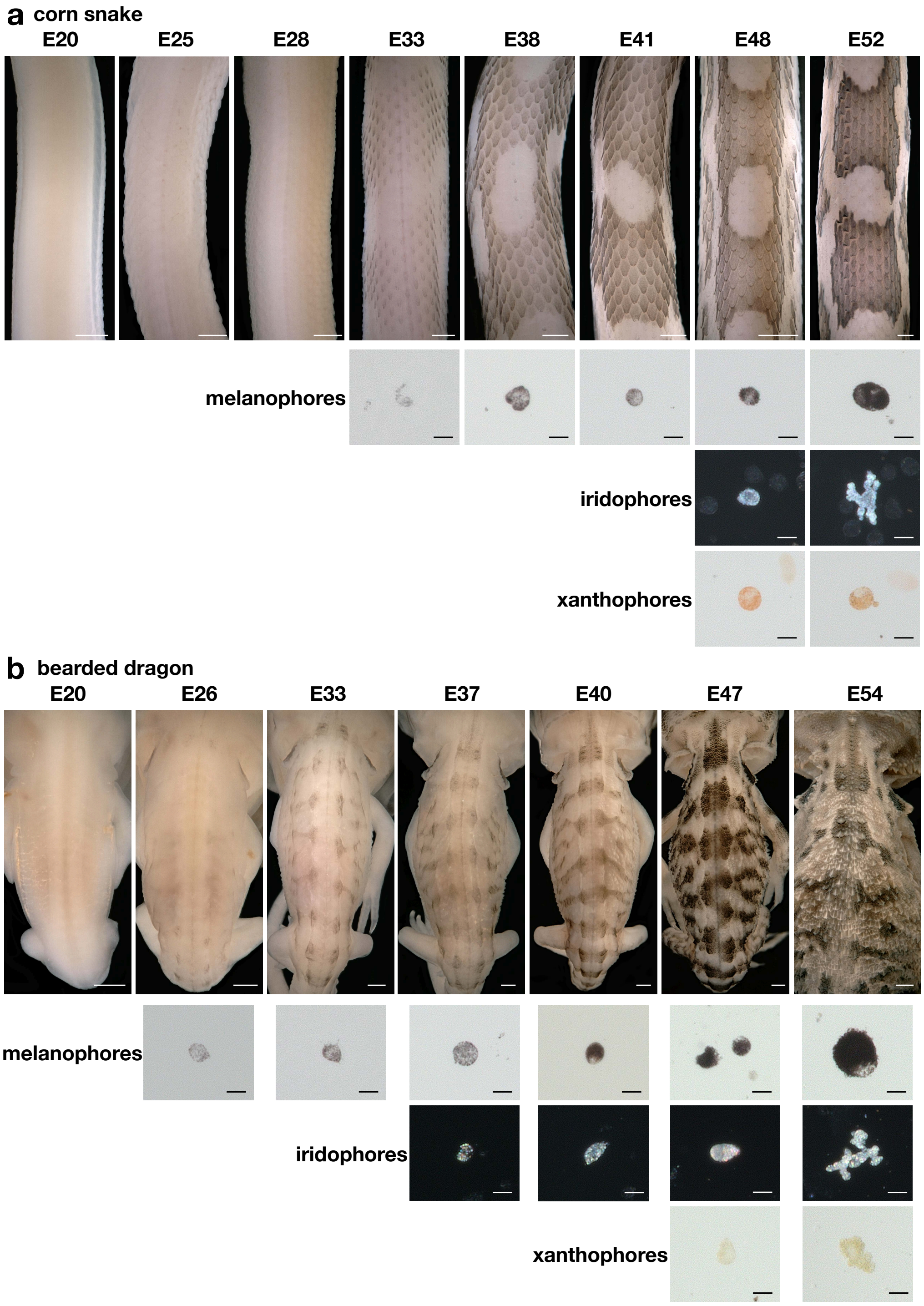


**Supplementary Figure 1. Corn snake and bearded dragon embryos and corresponding chromatophores** For the corn snake (**a**) and the bearded dragon (**b**), we provide photographs of fixed embryos at developmental stages corresponding to the single-cell RNA sequencing time points (top - scale bar = 1 mm), and microscopy images of the disassociated chromatophores present at each sampled stage (bottom - scale bar = 10 μm).

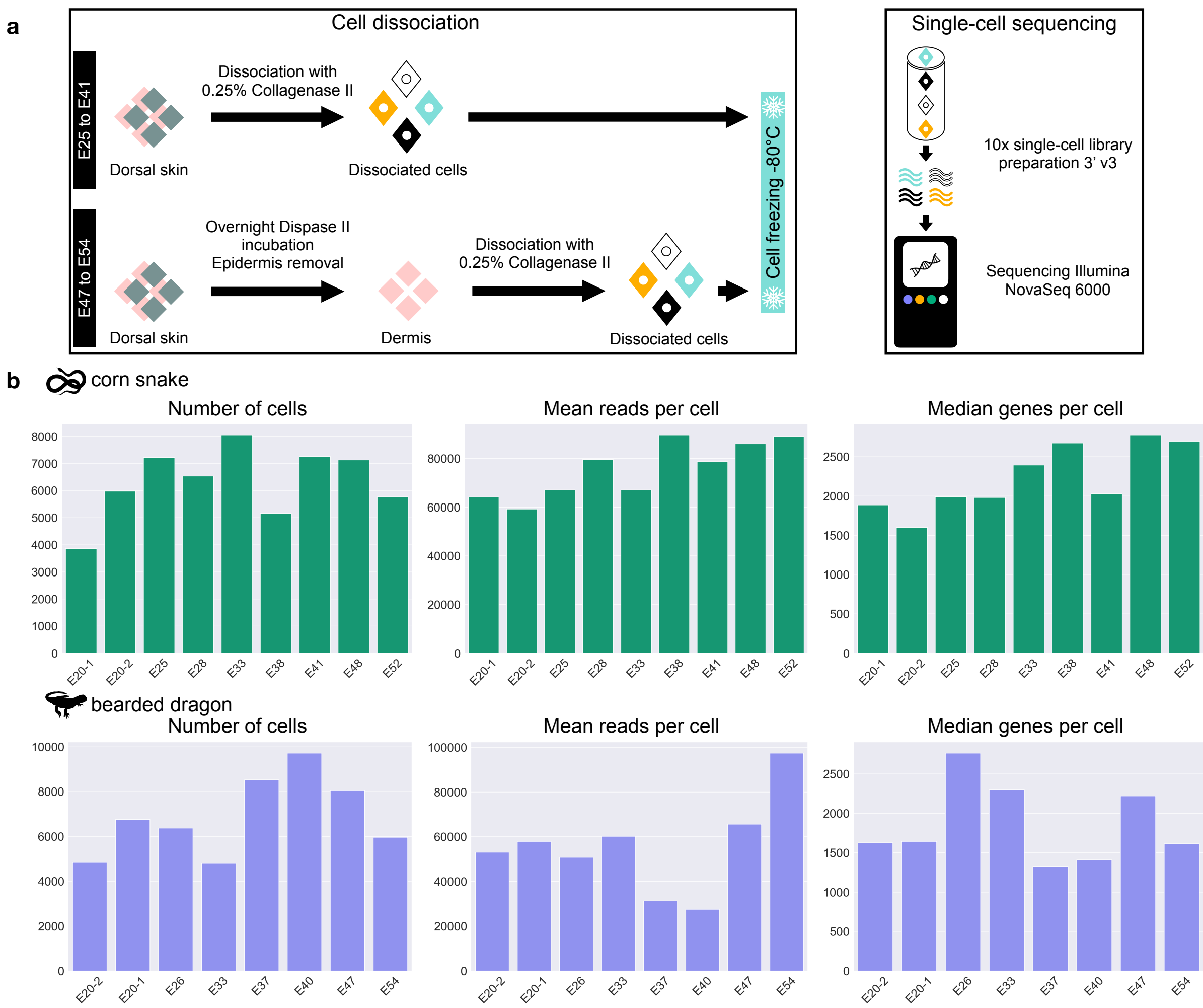


**Supplementary Figure 2. Protocol and quality metrics a.** Schematic overview of the experimental workflow. The left panel illustrates the tissue dissociation protocol, while the right panel depicts subsequent steps of RNA library preparation and sequencing. **b.** Cell counts and quality control metrics for corn snake (top) and bearded dragon (bottom) samples.

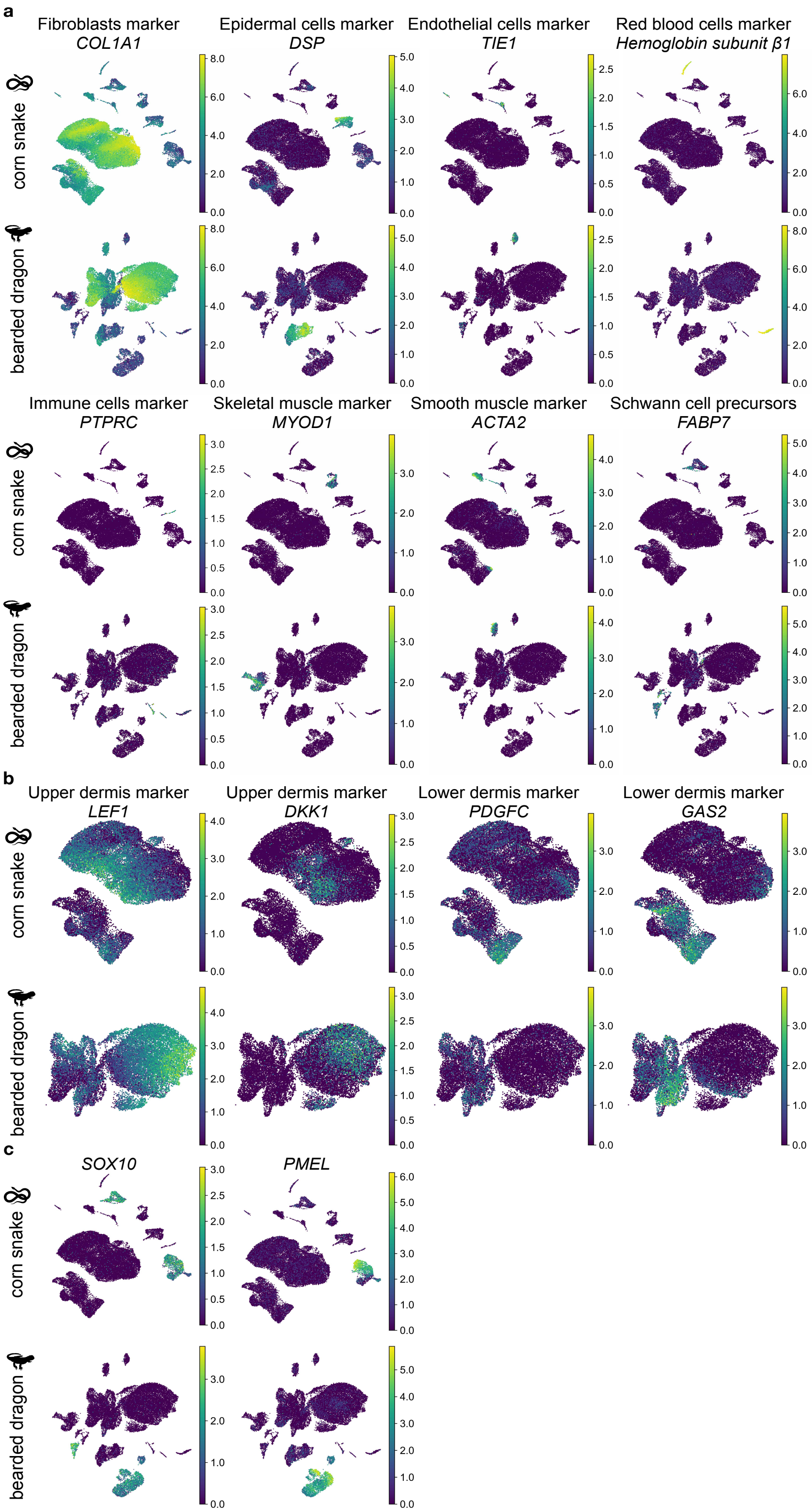


**Supplementary Figure 3. Skin cell type markers expression a.** UMAPs of the full datasets from corn snake and bearded dragon, showing the expression levels of representative cell type–specific marker genes. **b.** UMAPs of fibroblast clusters from corn snake and bearded dragon, showing the expression of markers specific to upper and lower dermis fibroblast subtypes as defined in Jacob et al., 2023. **c.** UMAPs of the full datasets showing the expression of *SOX10* and *PMEL*.

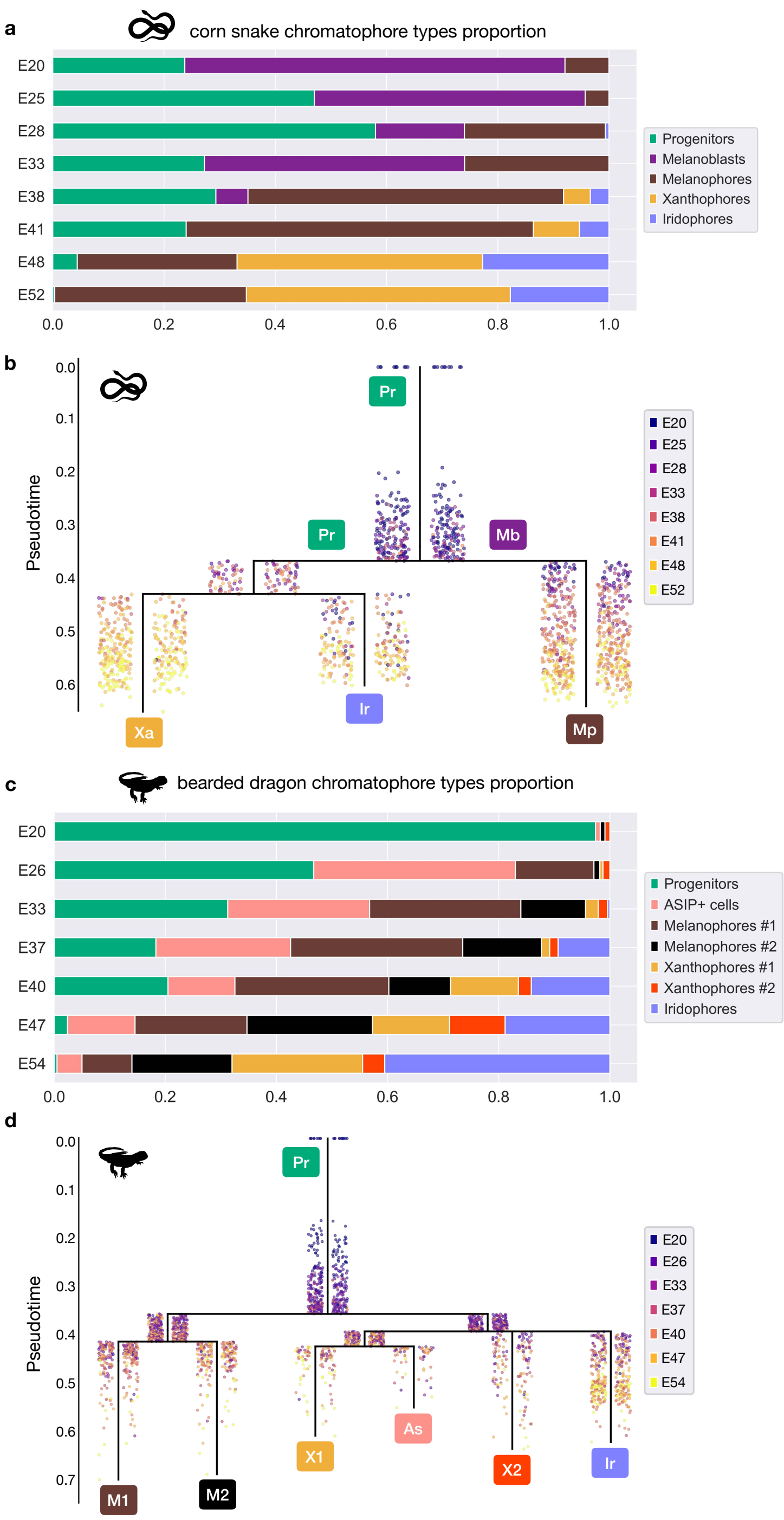


**Supplementary Figure 4. Cell populations and lineage trajectories across developmental time points in corn snake and bearded dragon a.** Proportion of each cell type cluster found at each stage sampled for corn snake (**a**) and bearded dragon (**c**). Trajectory tree inferred by URD tracing the progression from the root population to mature chromatophores for corn snake (**b**) and bearded dragon (**d**). Cells are coloured by stage sampled and positioned along the y-axis according to their assigned pseudotime. Branch points determined using preference divergence method, $p < 0.001$. Progenitors (Pr), Melanoblasts (Mb), Melanophores (Mp), Melanophores 1 (M1), Melanophores 2 (M2), Xanthophores 1 (X1), Xanthophores 2 (X2), Iridophores (Ir), and ASIP+ cells (As).

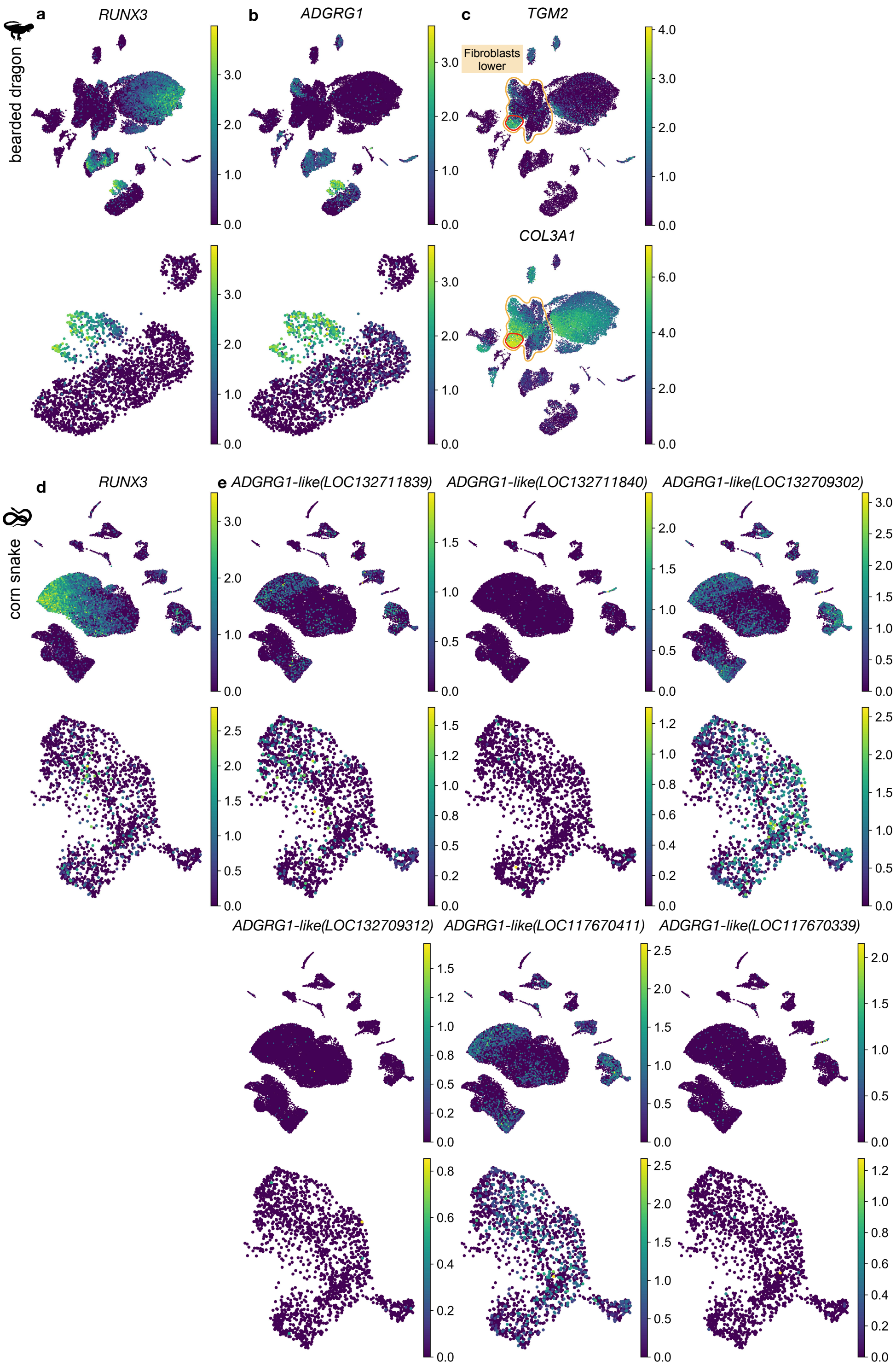


**Supplementary Figure 5. Expression pattern of secondary populations markers in bearded dragon and corn snake chromatophores a.** UMAPs showing *RUNX3* expression in bearded dragon entire dataset (top) and in chromatophores only (bottom). **b.** UMAPs showing *ADGRG1* expression in bearded dragon entire dataset (top) and in chromatophores only (bottom). **c.** UMAPs showing *TGM2* (top) and *COL3A1* (bottom) expression in bearded dragon entire dataset. The fibroblast lower cluster is delineated in yellow. UMAPs showing *RUNX3* (**d**) and *ADGRG1* orthologs (**e**) in the corn snake entire dataset (top) and in chromatophores only (bottom).

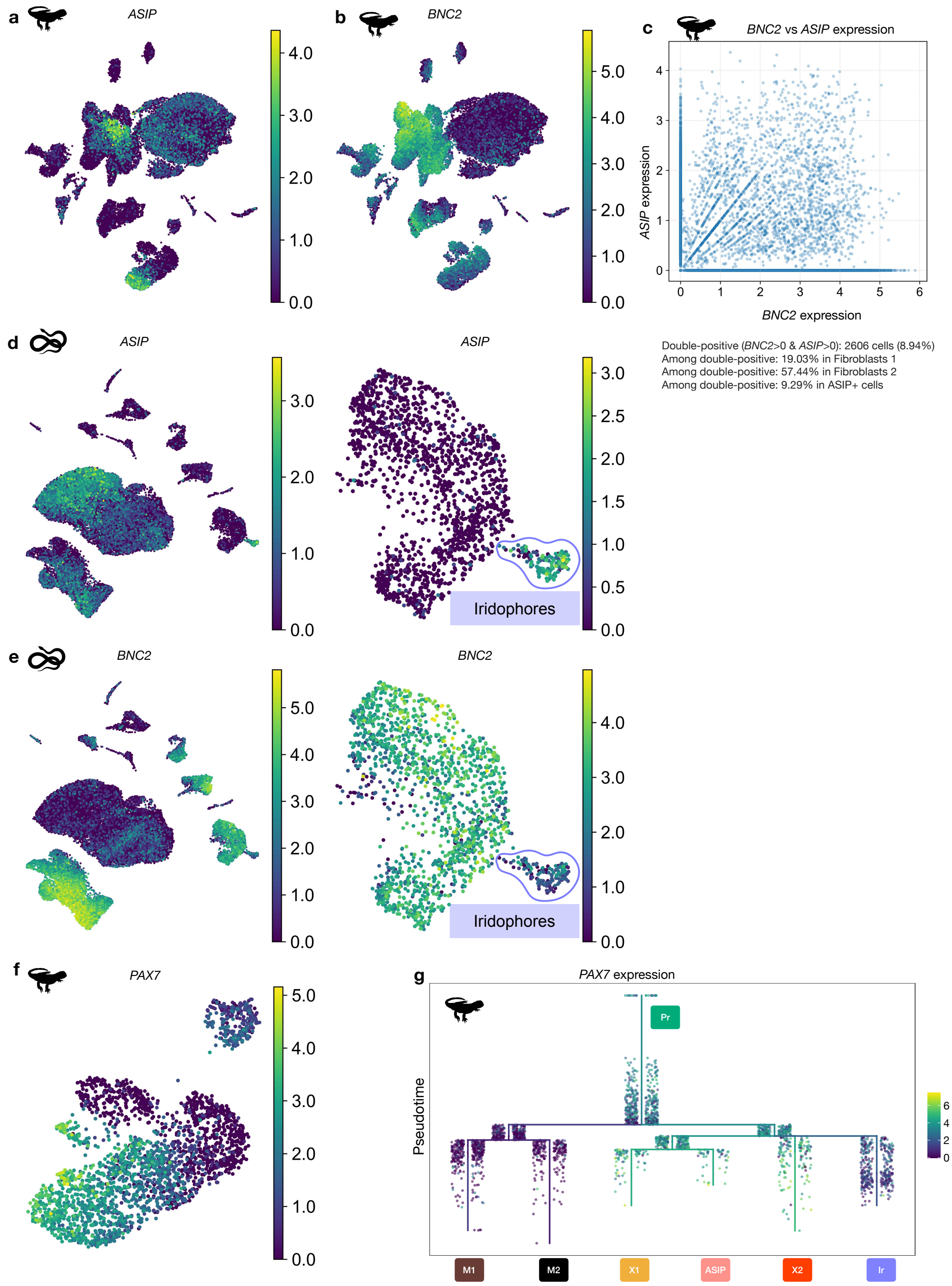


**Supplementary Figure 6. *ASIP*-expressing cells in corn snake and bearded dragon** UMAPs showing *ASIP* (**a**) and *BNC2* (**b**) expression in the bearded dragon entire dataset. **c.** Scatter plot of *BNC2* versus *ASIP* expression in individual cells from the bearded dragon entire dataset. UMAPs showing *ASIP* (**d**) and *BNC2* (**e**) expression in corn snake entire dataset (left) and in chromatophores only (right). The iridophores cluster is delineated. **f.** UMAP showing *PAX7* expression in bearded dragon chromatophores. **g.** URD-inferred trajectory tree for the bearded dragon, showing *PAX7* expression at single-cell resolution along the tree, as well as the average *PAX7* expression across individual branches.

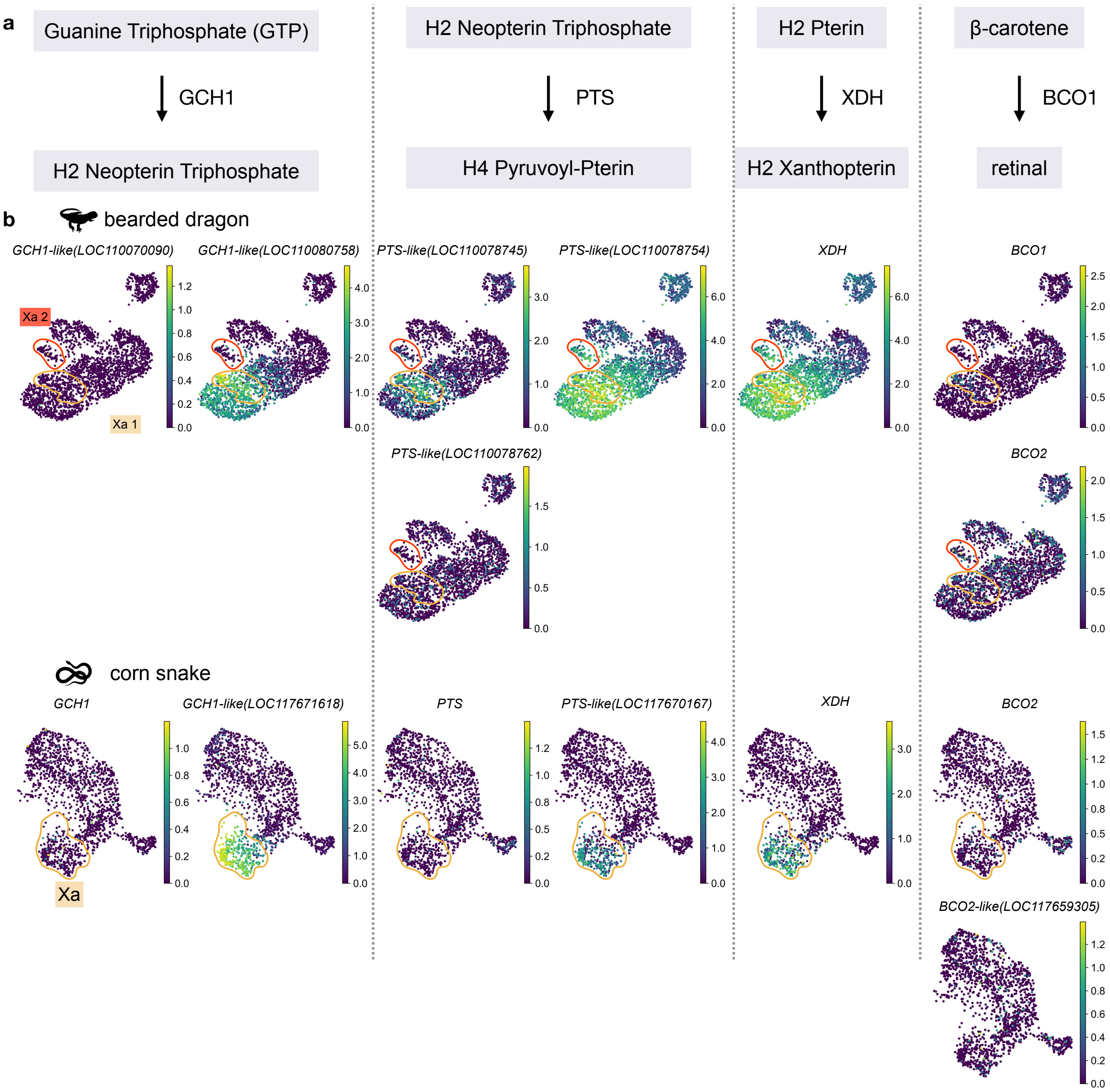


**Supplementary Figure 7. Expression of pteridine synthesis pathway genes in the bearded dragon and corn snake embryonic skin. a.** Schematic representation of key enzymatic steps in the pteridine synthesis pathway. Enzymes catalysing each reaction are indicated on the right of the corresponding arrows. **b.** UMAPs showing the expression of genes and their paralogs encoding the enzymes depicted in (a) in the bearded dragon (top) and the corn snake (bottom).

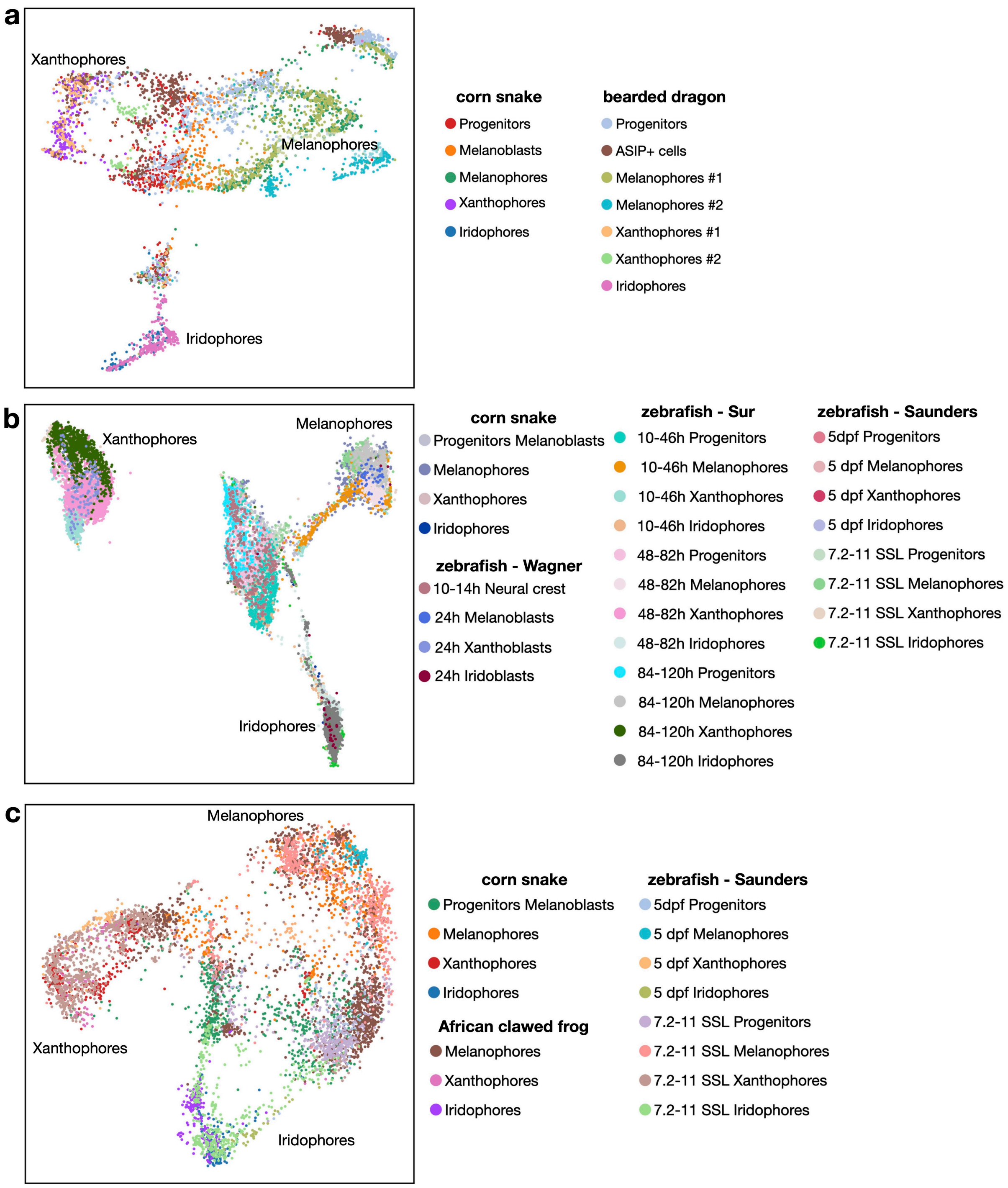


**Supplementary Figure 8. Dataset integration with SAMap. a.** UMAP of the SAMap-combined corn snake and bearded dragon datasets. **b.** UMAP of the SAMap-combined corn snake and zebrafish datasets. **c.** UMAP of the SAMap-combined corn snake, African clawed frog and zebrafish datasets. In all panels, each point is a cell embedded using the SAMap-integrated UMAP coordinates, and colors denote cell clusters for each species. The approximate regions corresponding to the three principal chromatophore lineages, i.e. melanophores, xanthophores, and iridophores, are indicated on the UMAP to highlight their overall distribution.

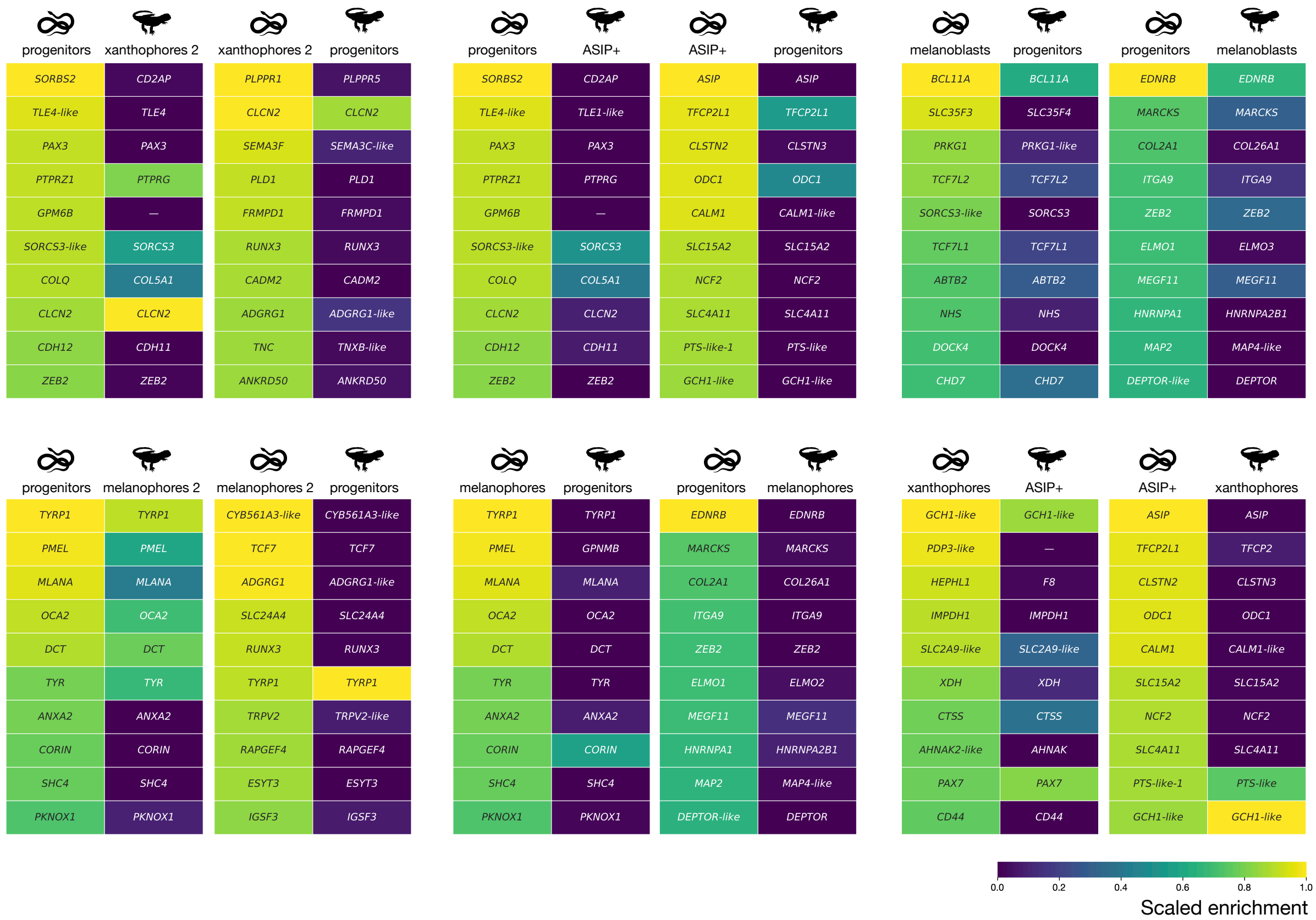

**Supplementary Figure 9. Cross-species comparison of highly differentially expressed genes in corn snake and bearded dragon cell clusters.** Comparison of differentially expressed (DE) genes in the chromatophore clusters of each species. For each type of cell, we provide the top DE genes in the corn snake and their homologs in the bearded dragon and vice versa. The heatmaps represent scaled enrichment. Only cluster pairs with an alignment score > 0.15 and not included in Figure 7b are shown. Enrichment was assessed using a two-sided Wilcoxon rank-sum test. Z-scores were normalized per cluster; negative values were set to zero.

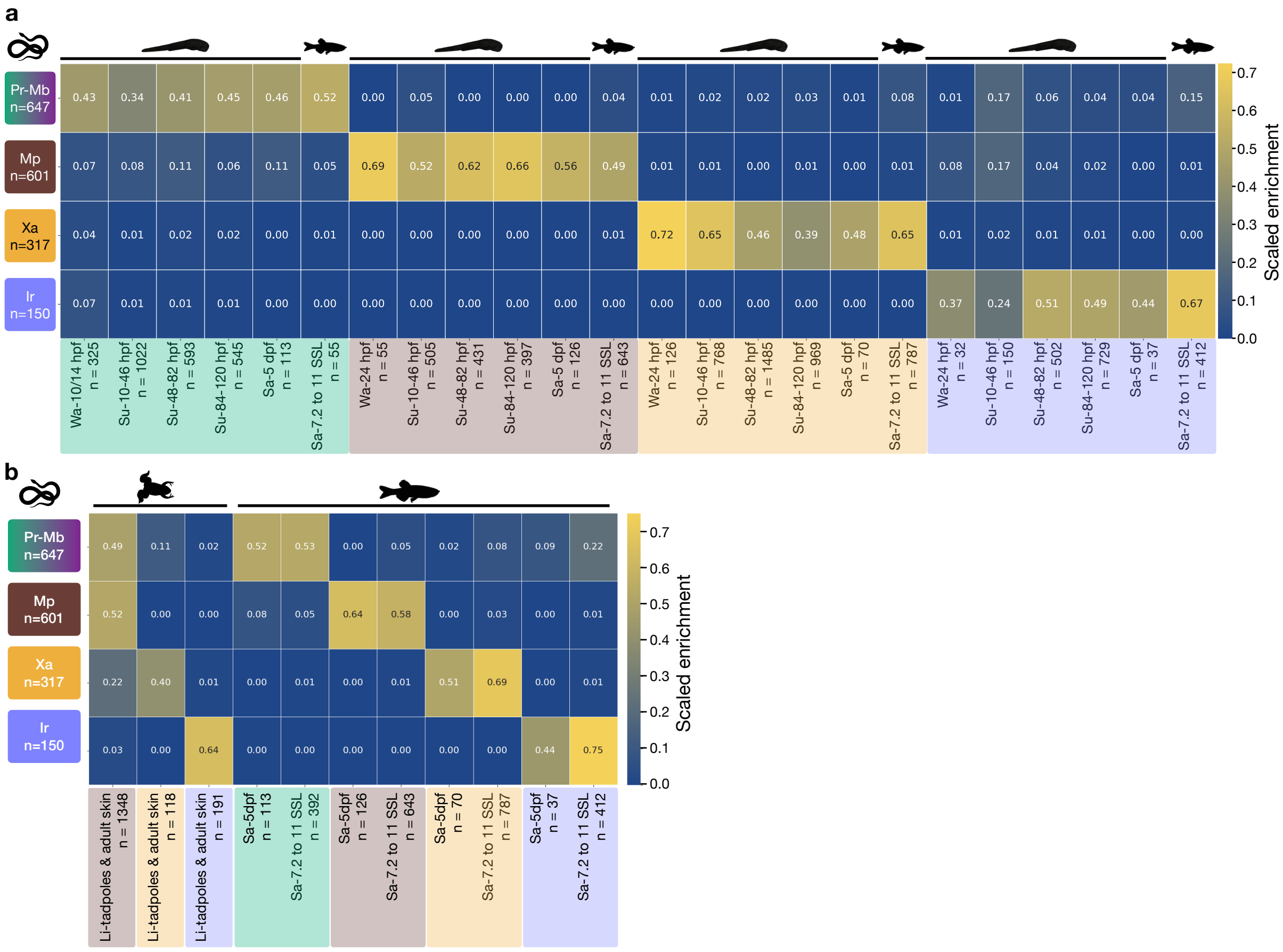


**Supplementary Figure 10. Cluster alignment scores for cross-species integrations a.** Heatmap of the cell type alignment scores between the corn snake and the zebrafish chromatophore clusters (zebrafish datasets Wa: Wagner et al., 2018; Su: Sur et al., 2023; Sa: Saunders et al., 2019). **b.** Heatmap of the cell type alignment scores between the corn snake, the frog (Li: Liao et al., 2022) and zebrafish (Sa: Saunders et al., 2019) chromatophore clusters. Colour code of clusters: green for progenitors, brown for melanophores, yellow for xanthophores, blue for iridophores.

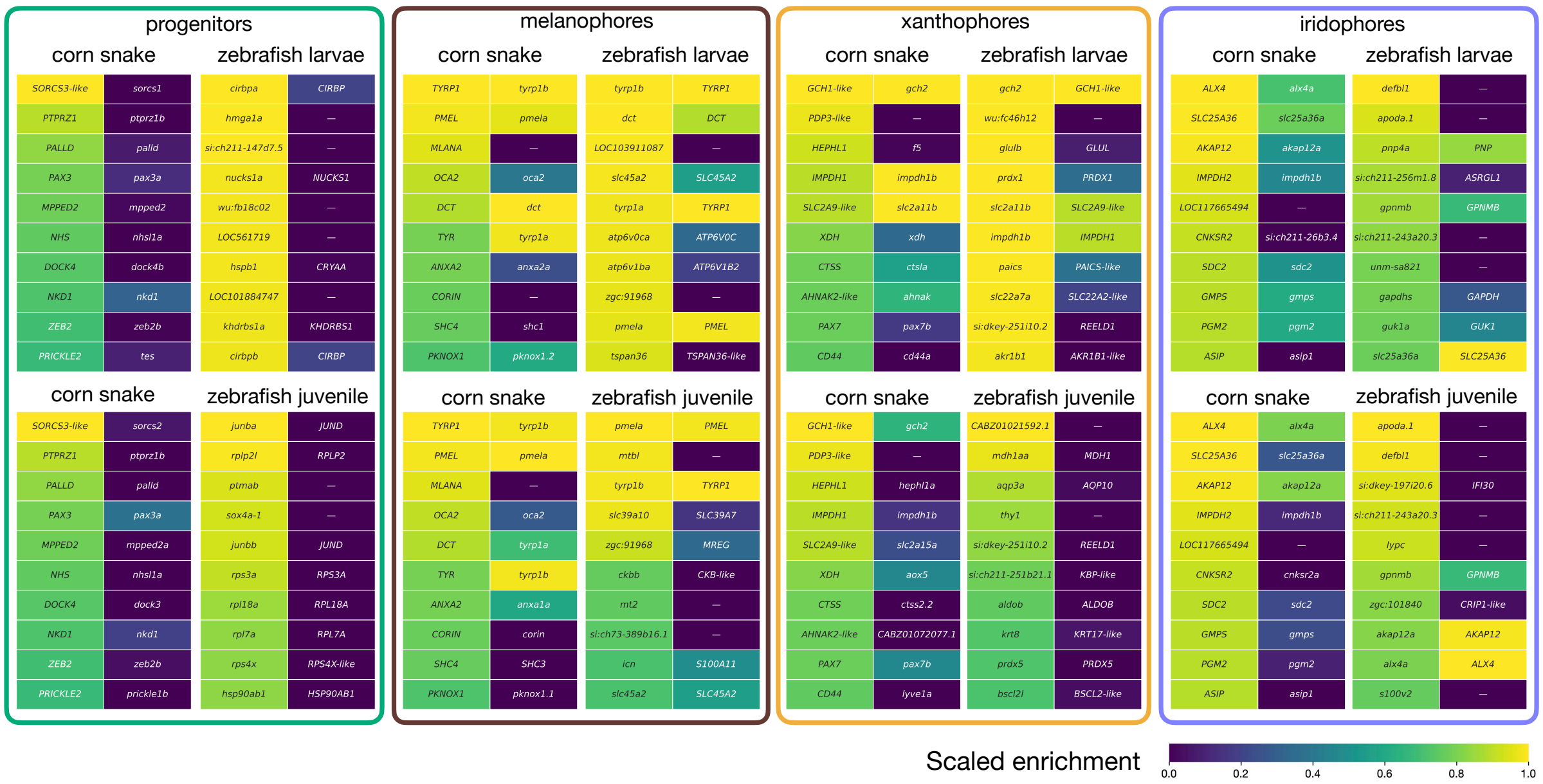


**Supplementary Figure 11. Cross-species comparison of top differentially expressed genes between corn snake and zebrafish chromatophores.** Comparison of differentially expressed (DE) genes in the chromatophore clusters of each species. For each type of cell, we provide the top DE genes in the corn snake and their homologs in the zebrafish and vice versa. The heatmaps represent scaled enrichment. Top marker comparisons were performed with both the embryonic and the juvenile/post-metamorphic stages, besides for iridophores. These chromatophores differentiate later so we used the larval instead of the embryonic stages. Enrichment was assessed using a two-sided Wilcoxon rank-sum test. Z-scores were normalized per cluster; negative values were set to zero.

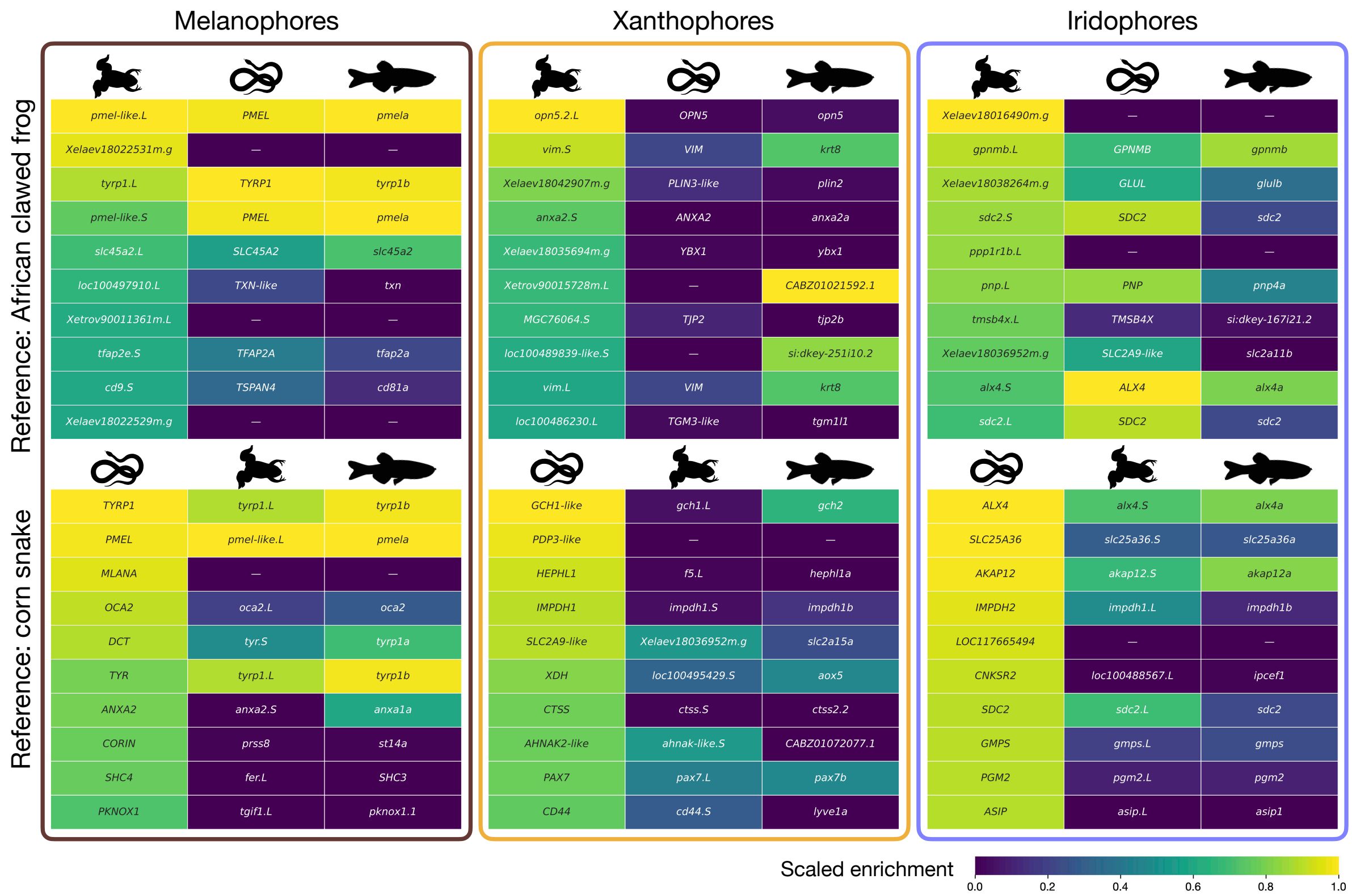


**Supplementary Figure 12. Cross-species comparison of top differentially expressed genes in chromatophores of the corn snake, the zebrafish and the frog (*Xenopus laevis*).** Comparison of differentially expressed (DE) genes in the chromatophore clusters of each species. For each type of cell, we provide the top DE genes in the corn snake and their homologs in the zebrafish and the frog. The heatmaps represent scaled enrichment. Enrichment was assessed using a two-sided Wilcoxon rank-sum test. Z-scores were normalized per cluster; negative values were set to zero.
