# Supplementary material for: Conserved and lineage-specific mechanisms drive chromatophore differentiation in reptiles
Source: Nat Commun. 2026 Jul 6;17:8368. doi: 10.1038/s41467-026-75281-5 (PMC13473439; doi:10.1038/s41467-026-75281-5)
Supplement: Supplementary file 4 [file 41467_2026_75281_MOESM2_ESM.pdf]

Description of Additional Supplementary Files

File name: Supplementary Data 1
Description: Top corn snake and bearded dragon dorsal skin clusters specific genes (pval_adj <= 0.05).

File name: Supplementary Data 2
Description: Top corn snake and bearded dragon chromatophore clusters specific genes (pval_adj <= 0.05).

File name: Supplementary Data 3
Description: Number of corn snake and bearded dragon embryos sampled per stage.

File name: Supplementary Data 4
Description: Cluster alignment scores between chromatophore clusters of different species calculated by SAMAP.

File name: Supplementary Data 5
Description: Ortholog mapping and enrichment summary for the integration of the corn snake, zebrafish and frog datasets.

File name: Supplementary Movie 1
Description: Three-dimensional force-directed layout of the URD-inferred developmental tree of chromatophore clusters in the corn snake. Each node represents a single cell positioned according to transcriptomic similarity and pseudotemporal ordering inferred by URD. Colors indicate chromatophore types: progenitors - green, melanoblasts - purple, melanophores - brown, iridophores - blue, xanthophores - yellow.

File name: Supplementary Movie 2
Description: Three-dimensional force-directed layout of the URD-inferred developmental tree of chromatophore clusters in the bearded dragon. Each node represents a single cell positioned according to transcriptomic similarity and pseudotemporal ordering inferred by URD. Colors indicate chromatophore types: progenitors - green, melanophores 1 - brown, melanophores 2 - black, ASIP^{+} cells - pink, iridophores - blue, xanthophores 1 - yellow, and xanthophores 2 - orange.
